# Embedment of sensing elements for robust, highly sensitive, and cross-talk–free iontronic skins for robotics applications

**DOI:** 10.1126/sciadv.adf8831

**Published:** 2023-03-03

**Authors:** Junli Shi, Yuan Dai, Yu Cheng, Sai Xie, Gang Li, Yuan Liu, Jingxiao Wang, Ruirui Zhang, Ningning Bai, Minkun Cai, Yuan Zhang, Yifei Zhan, Zhengyou Zhang, Cunjiang Yu, Chuan Fei Guo

**Affiliations:** ^1^ Department of Materials Science and Engineering, Southern University of Science and Technology, Shenzhen, Guangdong 518055, China.; ^2^ Tencent Robotics X, Shenzhen, Guangdong 518000, China.; ^3^ Department of Physics and TcSUH, University of Houston, Houston, TX 77204, USA.; ^4^ Department of Engineering Science and Mechanics, Pennsylvania State University, University Park, PA 16802, USA.; ^5^ Department of Biomedical Engineering, Pennsylvania State University, University Park, PA 16802, USA.; ^6^ Department of Materials Science and Engineering, Materials Research Institute, Pennsylvania State University, University Park, PA 16802, USA.; ^7^ Centers for Mechanical Engineering Research and Education at MIT and SUSTech, Southern University of Science and Technology, Shenzhen, Guangdong 518055, China.; ^8^ Guangdong Provincial Key Laboratory of Functional Oxide Materials and Devices, Southern University of Science and Technology, Shenzhen, Guangdong 518055, China.

## Abstract

Iontronic pressure sensors are promising in robot haptics because they can achieve high sensing performance using nanoscale electric double layers (EDLs) for capacitive signal output. However, it is challenging to achieve both high sensitivity and high mechanical stability in these devices. Iontronic sensors need microstructures that offer subtly changeable EDL interfaces to boost sensitivity, while the microstructured interfaces are mechanically weak. Here, we embed isolated microstructured ionic gel (IMIG) in a hole array (28 × 28) of elastomeric matrix and cross-link the IMIGs laterally to achieve enhanced interfacial robustness without sacrificing sensitivity. The embedded configuration toughens and strengthens the skin by pinning cracks and by the elastic dissipation of the interhole structures. Furthermore, cross-talk between the sensing elements is suppressed by isolating the ionic materials and by designing a circuit with a compensation algorithm. We have demonstrated that the skin is potentially useful for robotic manipulation tasks and object recognition.

## Teaser

A structure design of artificial skins achieves high sensitivity and high robustness, while suppressing crosstalk between sensors.

## INTRODUCTION

Flexible pressure sensors or electronic skins (e-skins) are devices that can transduce physical stimuli into electrical signals, and they are of great potential to change the robotic world by enabling robots to sense and percept the real world (*1*–*4*). Similar to the human skin, e-skins can provide abundant physical information for a robot during its interaction with the environment, human beings, or other robots (*5*–*9*). A desired e-skin for friendly robotic interaction needs to present human-skin–like softness and stretchability, high sensing performances, together with high mechanical stability.

A grand challenge for e-skins lies in the contradiction between their sensing properties and mechanical stability. Existing e-skins or flexible pressure sensors often have a layered device configuration (*10*–*12*). The most commonly used strategy to enhance sensitivity is introducing microstructures to the interlayer that enable a sensitively changed interfacial behavior (*1*, *13*–*15*). Such a layered device configuration with microstructured interfaces, however, are mechanically weak, and delamination or fracture of the device can easily occur upon shearing, in-plane compression, or bending (fig. S1 A and B). As a result, the devices can hardly survive in harsh mechanical conditions, such as robotic griping manipulation of heavy objects (which produces both high shear stresses and high compressive stresses).

Another challenge of e-skins is the difficulty to minimize pixels and to eliminate cross-talk between a large number of pixels (*16*). Traditional capacitive sensors have a low capacitance density (a typical value is 10 pF cm^−2^), which results in poor signal-to-noise ratio when the devices are minimized (*17*). For example, the signal magnitude is ~0.1 pF when the device area decreases to 1 mm^2^. Iontronic pressure sensors are a type of promising capacitive devices that can address the minimization issue using a soft ionic material to replace the regular dielectric layer, producing an electric double layer (EDL) at the ionic material-electrode interface with a charge separation of ~1 nm. The nanoscale charge separation leads to an ultrahigh capacitance density up to 10 μF cm^−2^ (or 100 nF mm^−2^) (*18*, *19*), allowing for high-quality signals in minimized sensors. By engineering microstructures either on the electrode or on the soft ionic conductor (*20*, *21*), it is possible to modulate the iontronic contact area (*A*) or EDL capacitance (*C*) to achieve exceptionally high capacitance-to-pressure sensitivity (fig. S2, A and B). In existing iontronic skins, however, all sensing elements share one ionic layer, causing severe cross-talk between pixels because of the motion of ions in the ionic conductor (fig. S3). Furthermore, the detection of the signal in a sensor array may also induce cross-talk. Thus, cross-talk in both cases needs to be suppressed in real applications.

Here, we report a strategy of embedding iontronic sensing element array in a rubbery matrix to achieve combined high sensitivity (>174 kPa^−1^ in 0.15 Pa to 400 kPa), high mechanical stability, dense and minimized pixels (28 × 28 pixels in a 10 cm by 10 cm area), and negligible cross-talk. The skin uses polydimethylsiloxane (PDMS) as a stretchable matrix, with each microstructured sensing element buried in an individual hole (totally 28 × 28 holes). The embedded configuration, featured as isolated cavities accommodating laterally bonded isolated microstructured ionic gels (IMIGs), results in a subtly changed iontronic interface for high capacitance-to-pressure sensitivity together with cross-talk–free signals. The cavities effectively toughen and strengthen the interface by pinning the cracks and by elongating the interhole walls to dissipate energy, allowing the skin to work stably under combined high shear (44 kPa) and high compression (200 kPa) over 10,000 cycles. In addition, we design a readout circuit for the sensor array and apply an algorithm to further reduce the detection-induced cross-talk. Real-time pressure mapping free of cross-talk and high-accuracy (99.5%) object recognition in a prosthetic hand–based system using the iontronic skin have been demonstrated.

## RESULTS

### Embedment configuration and fabrication of the iontronic skin

**Fig. 1.**
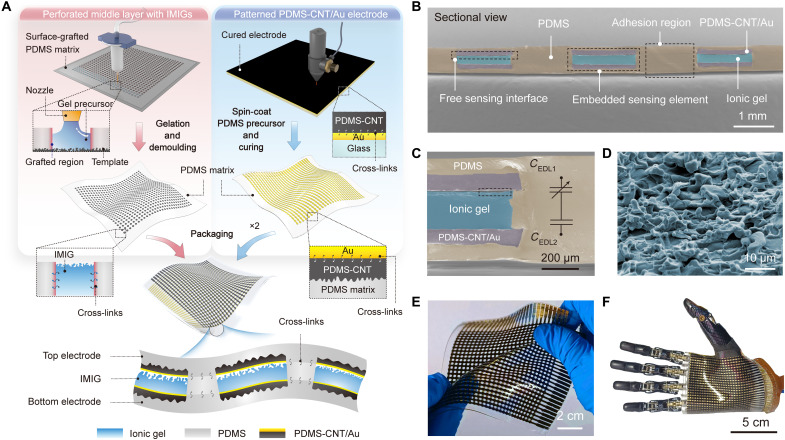
Fabrication of the iontronic sensor array (28 × 28) with embedded configuration. (**A**) Fabrication of the iontronic skin. The left column presents the fabrication of the perforated PDMS interlayer with embedded IMIGs; the right column presents the fabrication of the embedded electrode. Two electrode layers and the PDMS interlayer with embedded IMIGs in between were bonded together to integrate as a skin. (**B**) SEM image of the cross-sectional view of the sensor array. (**C**) Magnified view of the embedded sensing element and corresponding equivalent circuit. An air gap of ~10 μm between the electrode and microstructured ionic gel is observed, providing a sensitively changed sensing interface. *C*_EDL1_ represents the capacitance of the microstructured iontronic interface, and *C*_EDL2_ is the capacitance of the flat iontronic interface, which is fixed and large. (**D**) SEM image of the surface morphology of the IMIG, which corresponds to the black rectangular box in (C). (**E** and **F**) Optical photographs of the iontronic skin, showing that the skin can be greatly stretched. The high flexibility allows skin to be conformably laminated on the curved surface of prosthetic hand. CNT, carbon nanotube.

Unlike conventional designs for flexible pressure sensors or e-skins that use a loosely stacked, layered structure, our iontronic skin uses a seamlessly integrated configuration of which 28 × 28 sensing elements are embedded in a soft PDMS matrix (Fig. 1A). The diameter of a single pixel is 1.5 mm, and the interpixel distance is 2.8 mm, and the total area of the skin is 10 cm by 10 cm including wires. The PDMS matrix is integrated by intercross-linking of a trilayer, for which the middle PDMS layer is perforated to have a hole array to accommodate IMIG, as shown in the scanning electron microscopy (SEM) image of Fig. 1B. For the fabrication of the IMIG, ionic gel precursor is injected in the holes, with one surface of the ionic gel being templated using an abrasive paper to form microstructures and the side surface forming chemical cross-links with the side walls of the PDMS membrane (fig. S4). The middle layer with IMIGs is further encapsulated between two layers of patterned, PDMS-based electrodes (figs. S5 and S6) by forming cross-links between the trilayers, allowing the IMIG to form a changeable EDL interface (with microstructures) and a large and fixed EDL interface with the electrodes (Fig. 1C).

All materials and structures in this system are elaborately selected. We used polyvinyl alcohol (PVA):H_3_PO_4_, an ionic material for which electrical and mechanical properties can be precisely adjusted, as the IMIG. The PVA:H_3_PO_4_ IMIG is highly stretchable and exhibits a Young’s modulus of 1.6 MPa, comparable to that of the PDMS matrix (1.3 MPa) (fig. S7). In addition, the two electrodes are a PDMS-based composite that is made conducting by doping 7 weight % (wt %) carbon nanotubes (CNTs), followed by transferring a thin Au film on top of the composite. This electrode exhibits high electrical conductance, either in nonstrained or strained state (6.25 × 10^3^ S m^−1^ under 30% strain). The electrodes are also embedded in PDMS matrix by mechanical interlocking and placed on the top and bottom sides of the IMIG. The similar chemistry and mechanical properties enable robust adhesion between different functional layers of the devices. Furthermore, the IMIG has a “graded intrafillable architecture” (Fig. 1D), for which microscale protrusions buckle and fill into grooves and undercuts to avoid stress concentration and structural stiffening upon loading, providing a large structural compressibility that helps achieve high sensitivity in a wide pressure range (*13*).

The skin is highly stretchable because all components including the electrodes, the IMIGs, and the PDMS matrix are stretchable (fig. S7). Figure 1E and fig. S8 show that the sensor array can be stretched up to 150% without delamination or rupture, while the counterpart without bonded interfaces delaminates and rupture at a smaller strain. Different from most existing stretchable devices that achieve high stretchability by engineering structures of the electrodes, such as applying an island-bridge structure or kirigami (*22*, *23*), our sensor array is intrinsically stretchable, allowing for better conformability to integrate on curved surface (Fig. 1F).

### Toughening and strengthening effect of the embedded configuration

**Fig. 2.**
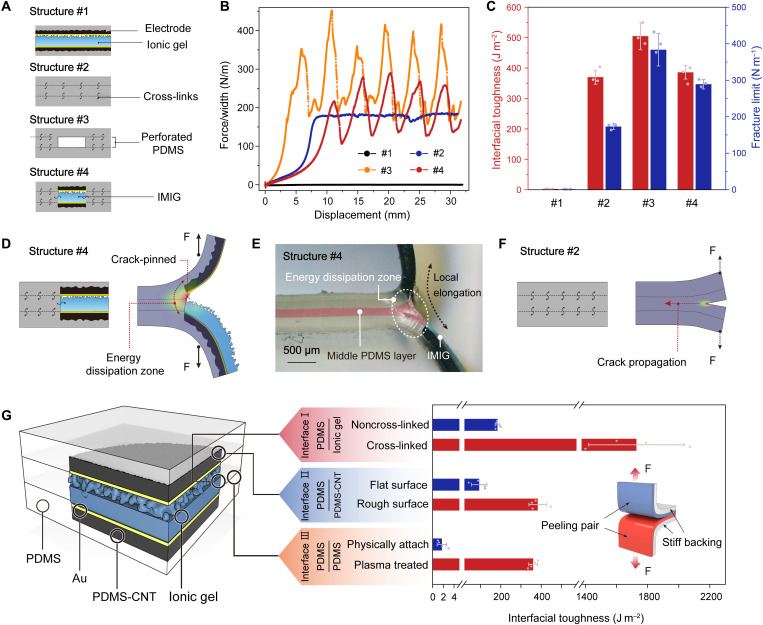
Interfacial mechanical properties the iontronic skin. (**A**) Schematic of four device configurations. (**B**) Peel force as a function of displacement for the four configurations. (**C**) Interfacial toughness and fracture limit of the four configurations. (**D**) Schematic showing the initial state and the state under peeling of structure #4. (**E**) Optical image showing that the interhole walls of structure #4 (our skin structure) is notable elongated, and the crack is pinned by the hole. (**F**) Schematic showing the initial state and the state under peeling of structure #2. (**G**) Schematic showing the interfaces of embedded sensing elements and the interfacial toughness values of the three interfaces indicated.

The embedded hole array has a toughening and strengthening effect that helps improve the mechanical stability of the iontronic skin. We illustrate the effect of the embedment configuration by comparing the interfacial toughness and fracture limit (or debonding resistance) of four configurations: (i) microstructured devices without interfacial bonding, (ii) a solid trilayer with bonded interfaces, (iii) a bonded trilayer with a perforated middle layer, and (iv) our embedded device configuration (Fig. 2A). We conducted 180° peeling test to measure the debonding resistance and toughness of the four structures, and the results are shown in Fig. 2 (B and C). Structure #1 is widely used in pressure sensors because it provides subtly changed interface and thus high sensitivity, but the device is mechanically poor (debonding resistance of ~0 and interfacial toughness of ~0). Structure #2 has poor response to pressure because the flat dielectric layer is incompressible while exhibiting mechanical properties identical to that of bulk PDMS (debonding resistance of ~173 N m^−1^ and interfacial toughness of ~370 J m^−2^). Structure #3 is evolved from structure #2 by replacing the middle layer with a perforated membrane. We find that the introduction of the cavities leads to a higher fracture resistance (384 N m^−1^) and higher toughness (506 J m^−2^) than that of the solid trilayer. Structure #4 is the combination of structure #1 and #3 made by filling up IMIGs in the cavities of structure #3. This structure exhibits a debonding resistance of 289 N m^−1^ and interfacial toughness of 386 J m^−2^, which are in between that of structure #2 and #3. Considering that the holes and the address lines (electrodes) in our skin (structure #4) occupy 49% of the total area, the true interfacial toughness is determined to increase by 105% compared with the bonded trilayer.

The toughening and strengthening effect of the skin (structure #4) stems from two aspects of the structure design. First, the hole array can hinder the propagation of cracks. A crack propagates catastrophically in a continuous elastomer, whereas in a patterned elastomer (e.g., with a hole array), a crack will be pinned by the interhole wall (Fig. 2D) until the peeling force exceeds the maximum adhesion force (or debonding resistance) to initialize a new crack. As a result, the peeling force shows a zigzag shape—it increases when the crack is pinned and decreases as a new crack is initialized. Note that the maximum peeling force is far higher than the case without holes, and such a strengthening effect can help stabilize the interface. Second, the interhole walls can elongate out of plane to dissipate strain energy (movie S1), serving as an elastic dissipator (*24*). The elastic dissipation can be verified in our optical microscopic observation, showing large local elongation of the middle layer that leads to substantial energy dissipation (Fig. 2E). By contrast, in solid PDMS (or a bonded PDMS trilayer, structure #2) that has no cavities, once a crack forms, it propagates rapidly and leads to catastrophic failure, and the energy dissipation zone is rather small because the crack tip is sharp (Fig. 2F). As a result, a lower toughness value is achieved in the solid material (or structure #2; fig. S9) than in the hollow structures (structure #3 and #4). Furthermore, if the material is not highly stretchable, then little energy can be dissipated, and the structured interface will not be toughened.

The thickness of the holes affects the strengthening effect: Structure #4 is less tough than structure #3 because the IMIGs in structure #4 thin out the holes. The smaller radius of curvature of the IMIG-filled holes in structure #4 leads to more severe stress concentration and thus a lower debonding resistance. As a result, the fracture limit (289 N m^−1^) of structure #4 is lower than that of structure #3 (384 N m^−1^) but nearly twice the fracture force of bulk PDMS (173 N m^−1^). When the load (peel force per width) is below 289 N m^−1^, crack propagation along the hollow structure is not possible, while at a load between 173 and 289 N m^−1^, failure of structure #2 occurs. Therefore, the embedded sensor configuration improves the stability of the devices, given that an improved interfacial toughness and a much higher fracture limit are both observed.

The toughening and strengthening effect require strong adhesion between different layers. In our skin (structure #4), all the three PDMS layers are covalently bonded together. The IMIGs are also laterally merged with the perforated PDMS interlayer by cross-linking (fig. S4), while the top surface is free to sensitively respond to normal pressure. The formation of lateral cross-links between PDMS and PVA chains is critical. Cross-links cannot be directly formed between PDMS and PVA. Here, the side wall of the PDMS holes was plasma-treated to introduce dense Si-OH groups, followed by the sequential immersion in two solutions of hydrolyzed aminopropyltriethoxysilane (APTES) and glutaraldehyde (GA). The Si-OH groups suspended on the PDMS surface condensate with the hydrolyzed APTES, and the amino group at the other end of the APTES molecules can connect with an aldehyde group of the GA molecules through the Schiff base reaction, while the other aldehyde group of GA reacts with the oxhydryl groups of the PVA chains, forming covalent bonding between PDMS and PVA.

Interfacial toughness values of three interfaces, including the chemically cross-linked interface between the IMIG and the perforated PDMS layer (denoted as interface I), the interlocked interface (see fig. S10) between the electrodes and the PDMS matrix (interface II), and the covalently bonded interface between different layers of the PDMS matrix (interface III) are determined to be 1730, 380, and 360 J m^−2^ (Fig. 2G), respectively, indicating that all these interfaces are highly stable. Without cross-links or mechanical interlocks, the toughness values of the three interfaces markedly decrease to 184, 93, and 2 J m^−2^, respectively, and interfacial debonding can easily occur upon peeling because the values are all lower than the fracture toughness of PDMS.

### Sensing properties of the iontronic skin

**Fig. 3.**
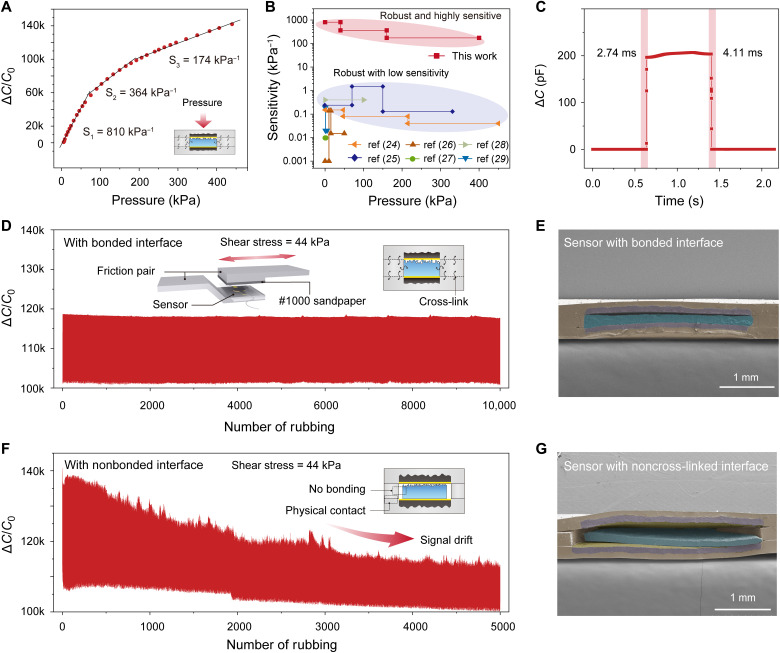
Sensing properties and stability. (**A**) Normalized change in capacitance as a function of pressure of the sensor. (**B**) Sensitivity as a function of pressure for our sensor and its comparison with existing sensors that have robust interfaces. Our sensor exhibits at least two orders of magnitude higher sensitivity over a wide pressure range (*24*–*29*). (**C**) Response and relaxation time measured by loading-unloading a pressure of 100 Pa. (**D**) Responses of the iontronic skin under cyclic rubbing (10,000 cycles) with combined shear (44 kPa) and pressure (200 kPa). Inset shows the schematic of the setup for the rubbing test. The friction pair consists of a piece of #1000 abrasive paper and the sensor. (**E**) Cross-sectional view SEM image of the skin after the rubbing test. No delamination was observed. (**F**) Response of rubbing test for a control sample with non–cross-linked interfaces under the same testing condition of (D). Inset shows the sensor structure. (**G**) SEM image showing that the sensor is damaged during the rubbing test.

The embedment configuration enables combined high robustness and high sensing properties. The sensitivity of capacitive sensors is defined as δ(Δ*C*/*C*_0_)/δP, where *C* and *C*_0_ are the instantaneous capacitance and initial capacitance before loading and *P* is the applied pressure. We measured the Δ*C/C*_0_-*P* curve of a single sensor and calculated its sensitivity values in different pressure ranges. The sensitivity is 810 kPa^−1^ within 40 kPa and drops to 364 kPa^−1^ in 40 to 160 kPa and lastly maintains at 174 kPa^−1^ in 160 to 440 kPa, as shown in Fig. 3A. This ultrahigh sensitivity over a broad pressure range is at least three orders of magnitude higher than that of existing capacitive sensors with bonded interfaces (Fig. 3B) (*24*–*29*), allowing the sensor to be used for the monitoring of weak physiological signal such as fingertip pulse (fig. S11).

The high sensitivity is related to the structure of the sensor. First, the microstructures of IMIG provide a large change in EDL interfacial area upon loading, in addition to improved compressibility that helps achieve a broad working range (*13*). Second, the embedment design elaborately introduces a thin air gap between the top electrode and the IMIG to have a fixed and small initial capacitance, which also contributes to large sensitivity.

The skin also exhibits a low response to shear stress together with a high response-relaxation speed. The interhole walls in the middle layer limit the lateral displacement between the electrode and the ionic material, resulting in poor response to shear stress, as shown in fig. S12. Such a property helps reduce the interference of static shear to normal pressure. On the other hand, the structure improves the response-relaxation speed of the skin. Figure 3C shows that the sensor exhibits a rapid response time of 2.74 ms and a recovery time of 4.11 ms, which are about one order of magnitude shorter than that of the human skin and existing iontronic sensors (*30*–*32*). The rapid response-recovery speed is contributed by the abundant microstructures of the IMIG that can results in a high energy release rate (*15*), as well as the sealed air gap that serves as an “air spring” for rapid recovery. Because of the rapid response-relaxation speed, this sensor can detect high frequency vibrations up to 120 Hz (fig. S13).

We further tested the stability of a single sensing element under harsh mechanical conditions, and both the stability of the signal output and the sensor structure were evaluated. The sensor was first subjected to simple compression-release cycles with a peak pressure of 200 kPa, and negligible degradation in signal magnitude was observed after 10,000 loading-unloading cycles (fig. S14). In real applications, the stability of the skin under combined shear and compression is important. Therefore, the sensing element under a more complex condition, for instance, repeated rubbing under a load of 200 kPa (which generates a shear stress of ~44 kPa) at a speed of 5 mm min^−1^ over 2 mm for 10,000 cycles, was further tested. The sensor exhibited a highly stable signal output in such a harsh condition (Fig. 3D), and no damage in sensor structure (e.g., delamination or rupture) was observed in the SEM inspection (Fig. 3E). We ascribe the structure stability to the robust interfaces of the device. In contrast, a control sample without cross-linking the IMIG with the PDMS matrix shows substantial signal drift during the repeated compression-rubbing test (Fig. 3F). Accordingly, the sensor structure was fully damaged, as verified in our SEM observation (Fig. 3G).

### Cross-talk suppression using the IMIG and readout circuit design

**Fig. 4.**
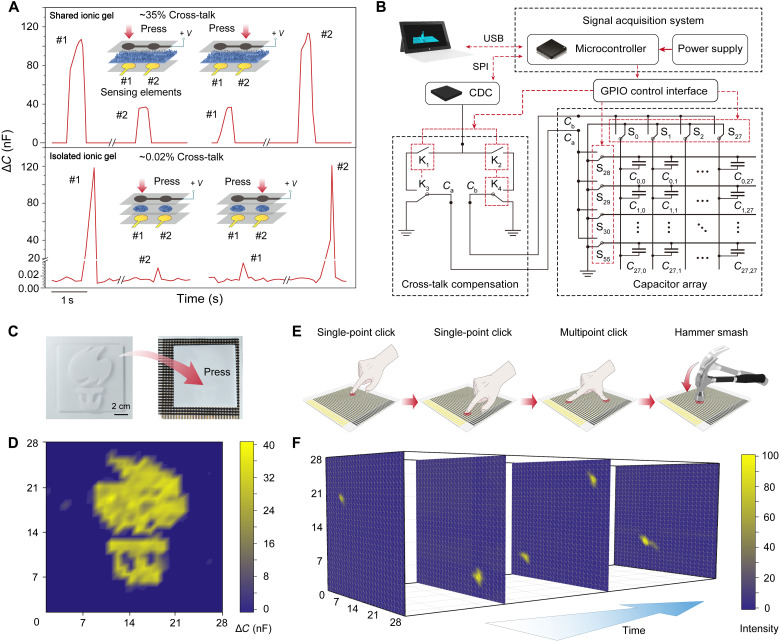
Cross-talk suppression and the signal acquisition system for pressure mapping. (**A**) IMIG design for cross-talk suppression. When using a shared ionic layer and pressing one of the two sensing elements, the neighboring sensing element will generate a signal with a magnitude 35% that of the one under test. When using the IMIGs, only 0.02% cross-talk is observed. (**B**) Diagram of the readout circuit, which has a cross-talk compensation module. Here, SPI and GPIO represent the serial peripheral interface and the general-purpose input output, respectively. (**C**) and (**D**) Signal mapping of the iontronic skin when a 3D-printed patten is covered on applying 5-kPa static pressure. (**E**) Schematic of dynamic stimuli including single-point touch, multipoint touch, and hammer smashing. (**F**) Dynamic signal mapping of the dynamic process using the signal acquisition system.

The IMIG design can effectively suppress the signal cross-talk, which is a commonly seen problem in iontronic sensor array using a shared ionic layer. Cross-talk gives incorrect or misleading information of the physical stimuli and needs to be prevented. Here, we found that the cross-talk can be well suppressed by isolating ionic materials in different sensing elements. We fabricated two sensing elements with IMIGs and two control sensor elements using a shared microstructured ionic gel (Fig. 4A). In both cases, the two sensing elements share a top electrode. For the two sensors using the IMIG, only ~0.02% cross-talk (defined as the signal amplitude of the neighboring sensor in reference to the one under loading) (*33*) was observed, which can be ignored. The cross-talk suppression can also be reflected in the overlapped Nyquist plots indicating that there is no ion migration between neighboring sensing elements (fig. S15) (*34*). For the control sample, however, the cross-talk is notable because of ion transfer, and a 35% giant cross-talk is observed. The large cross-talk indicates that the device configuration with a shared ionic layer can hardly be practically used.

The signal detection of a sensor array also induces cross-talk. In this work, a “row and column” configuration for the sensor array was used to reduce the number of address lines. The pixels that share the same address line are connected in parallel, and a signal induced by the detection is found, even in the case without touching any sensing element (see the mechanism of cross-talk in fig. S16). This detection-induced cross-talk needs to be further suppressed.

A readout circuit with the consideration of cross-talk suppression was made to detect the capacitance change of the pixels. Figure 4B shows the readout circuit diagram of the signal acquisition system. The sensor array was connected to the 28 rows and 28 columns of single-pole, double-throw (SPDT) analog switches via a flexible printed circuit connector. The analog switch scans the pixels in the sensor array one by one and shields all other sensing elements to the ground. A cross-talk compensation module based on the analog switch and the switching circuit was designed to suppress the cross-talk of the sensing elements in the same row or the same column. Detailed information of the cross-talk compensation module can be seen in figs. S17 to S19. With the cross-talk suppression, the degree of detection-induced cross-talk decreases from 75 to 0.67%. A microprogrammed control unit (MCU) that controls all analog switches was used to select the target sensing element and further switched different circuit states through the cross-talk compensation module. A capacitance-digital-converter (CDC) unit was used to detect the capacitance change under corresponding compensation circuit and send data to the MCU to compensate for the true capacitance of the sensing element.

### Applications of the iontronic skin

We demonstrate that the iontronic skin is capable of mapping the pressure distribution during either static or dynamic loading conditions because intercell cross-talk is suppressed. For the detection of static pressure, a resin model printed with a pattern was placed on the sensor array, and static capacitance values of all pixels were recorded. It shows that the pattern of capacitance signal matches well with the pattern of the printed model (Fig. 4, C and D), with little noise being observed. The skin can also be used for dynamic pressure mapping. In Fig. 4 (E and F), we show that when a volunteer touches different locations of the skin with a single finger and then two fingers, a real-time visual interface can display the dynamic pressure distribution of the process. Soon after that, the skin is smashed using a hammer, and the output is also recorded (movie S2), while no damage to the skin is observed.

**Fig. 5.**
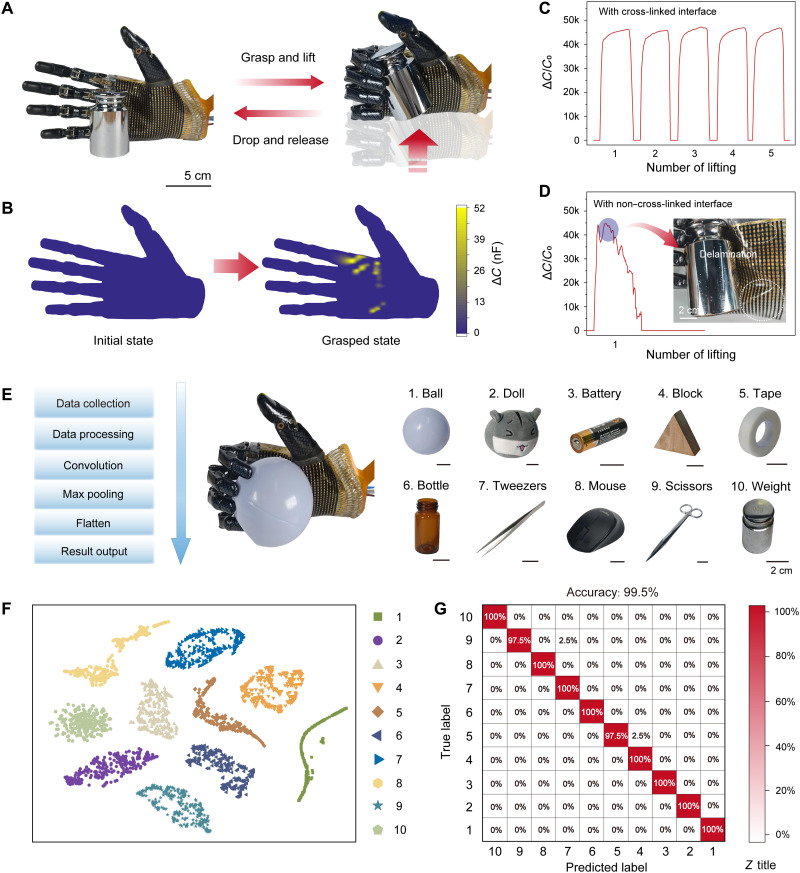
Application of the iontronic skin in robotics. (**A**) Prosthetic hand equipped with the iontronic skin for the grasping of a 500-g weight. The weight is grasped, lifted up to 20 cm, and lowered down to the initial height and released. (**B**) Signal mapping corresponding to the initial state and the lifted state. (**C**) Signal from one randomly selected sensing element in the repeated process of (A). (**D**) Signal of one randomly selected pixel from a control sensing array using a shared ionic layer for all sensing elements (without the embedded configuration and interlayer cross-linking). Inset demonstrates the delamination of the control sample. (**E**) Process of object recognition by machine learning. Ten different objects are used for the classification. (**F**) t-SNE visualization of clustered data. (**G**) Confusion map of the 10 tested objects, showing a recognition accuracy of 99.5%.

The sensor array can be used for real-time pressure detection in robotic manipulation. We adhered the sensor array to the palm of a prosthetic hand, which has a curved surface, to grab different objects, and the pressure distribution of the palm can be imaged in real time. When grasping a weight of 500 g, it generates a high shear stress up to ~10 kPa (Fig. 5, A and B), and the signal recorded from a randomly selected pixel is highly stable in a few cycles of gripping, lifting, and release of the weight (Fig. 5C). In contrast, a control sample for which the IMIG is not cross-linked with the PDMS matrix shows irregular signal output in the first cycle of loading, and no response is detected in subsequent cycles of manipulation (Fig. 5D). Accordingly, delamination of the control sample is observed (inset of Fig. 5D). The results verify the effectiveness of the interfacial cross-linking and embedded configuration in our iontronic skin to achieve high stability under harsh mechanical conditions.

The sensor array can be used not only for transducing force or pressure but also for the recognition of objects that a prosthetic hand grasps. The sensor array was adhered to a prosthetic palm for the detection of pressure distribution during the grasp of 10 different objects (including a plastic ball, a doll, a dry battery, a building block with a triangular shape, a roll of tape, a glass bottle, a pair of tweezers, a mouse, a pair of scissors, and a weight of 500 g). A deep learning model of one-dimensional convolutional neural network (1D-CNN) was trained to classify the grasped objects (Fig. 5E). Deep learning has been proven to be effective to automatically learn the features of data from pressure sensors (*35*–*37*). Here, we collected the data from a submatrix of 7 × 7 sensing elements to simplify the learning process. We recorded 300 sets of data for each pixel, among which 220 sets was used for training, 40 sets for validation, and the rested 40 sets for testing. After data collection, the *t*-distributed stochastic neighbor embedding (t-SNE) technique was used to reduce the dimensions of the data before learning is conducted. Visualization results are shown in Fig. 5F, indicating that the datasets from different objects are clustered together, as a result of distinguishable characteristics of different objects. The 1D-CNN model gives an average classification accuracy of 99.5%, as shown in the confusion map of Fig. 5G. In addition, the recognition can also be extended to a real-time system (movie S3). Such a high recognition accuracy offers great promise of the iontronic skin to be applied to the control and feedback in robotic manipulation tasks. Furthermore, it is expected that when more sensing elements are included for the object recognition, a higher accuracy can be achieved because it provides more representative features from the objects.

## DISCUSSION

Our iontronic skin is promising in robotic haptics and other applications because of its combined high performances. Whereas traditional designs for flexible pressure sensors can hardly achieve both high sensitivity and high mechanical robustness (*24*, *26*, *27*, *38*–*41*), our design of using embedded IMIGs enables combined high sensitivity (>174 kPa^−1^ in 0.15 Pa to 440 kPa) and high mechanical robustness (fracture limit: 289 N m^−1^ and interfacial toughness: 386 J m^−2^), in addition to skin-like softness and high stretchability (elongation at break >150%). In addition, our iontronic skin is made with a large area and high sensor density (28 × 28 pixels or ~8 pixels cm^−2^), close to the density of the mechanoreceptors in the human skin of the trunk. Iontronic sensor arrays often suffers from cross-talk, and here, we conducted cross-talk suppression by the isolation of ionic material and also by the circuit design and algorithm. The combined high performance of the skin may arouse a wide range of applications. For example, the wide-range high sensitivity is expected to enable tactile feedback for high-load manipulation tasks, plantar pressure detection of prosthetics, and pressure detection for aviation industry. Furthermore, the large-area and high-density array with precise force transduction make the applications of the skin in virtual reality possible.

Our work illustrates a principle of the structure design: Both interfacial toughness and strength can be enhanced by elaborately introducing embedded and isolated cavities (kirigami or other patterns) in the interlayer of a fully integrated skin, and this can be used as a general strategy to improve the mechanical stability of the skin. This design, on the other hand, effectively suppresses the cross-talk between the sensing elements while maintaining high sensing properties of the devices. We expect that the strategy of embedding sensing elements in a soft matrix to be widely extended to many other devices.

## MATERIALS AND METHODS

### Preparation of IMIGs

Commercial PDMS membrane with a thickness of 200 μm (Bald Advanced Materials Co. Ltd.) was used as the middle PDMS layer. The PDMS layer was perforated to have the hole array (diameter: 1.50 mm, interhole distance 2.77 mm) using a laser cutter (WE-6040, Beyond Laser Co. Ltd.). The side wall of holes was then treated using air plasma (TS-PL05, Dongxingaoke) at 50 W for 20 s. Next, the treated PDMS layer was immersed into the solution of 10 volume percent (volume %) APTES (99% in purity, MERYER), 45 volume % ethanol (AR, HUSHI), and 45 volume % deionized water under 40°C for 10 min. After cleaning with ethanol and deionized water sequentially, the aminated PDMS was immersed in 2.5 volume % GA (25 volume % in water, MAKLIN) aqueous solution at 40°C for 30 min, followed by washing in ethanol and deionized water and drying with N_2_ gas. Next, the surface-treated PDMS layer was affixed on a piece of abrasive paper (#10000, Eagle & AX), which serves as the template for the IMIG. Next, the ionic gel precursor, a solution of PVA and H_3_PO_4_ (10 volume % PVA:H_3_PO_4_ aqueous solution), was injected into the holes using a 3D printer (Bio-Architect SR, Regenovo Biotechnology Co. Ltd.) to form covalent cross-links between PDMS and PVA chains. After gelation of PVA:H_3_PO_4_, the abrasive paper was removed.

### Preparation of the stretchable electrodes and sensors with IMIG

A 100-nm Au film was deposited on the surface of a piece of smooth glass using electron beam evaporation (TF500, HHV). A solution, consisting of 10 volume % mercaptopropyl trimethoxysilane (95% in purity; Aladdin), 45 volume % ethanol, and 45 volume % deionized water, was used for the modification of the Au film. The pH value of the solution was modulated by adding glacial acetic acid (99.5% in purity; Shanghai Lingfeng Chemical Reagent), and then, a piece of glass was immersed into the solution. PDMS (Sylgard-184, Dow Corning, with a base to curing agent weight ratio of 10:1) and multiwall CNTs (7 wt %; XFM13, XFNANO) were added in chloroform (99% in purity; Shanghai Lingfeng Chemical Reagent) and dispersed in an ultrasonic homogenizer (JY92-IIN, SCIENTZ) for 2 hours. The PDMS-CNT dispersion was poured onto the modified Au film for solvent evaporation, followed by curing at 80°C for 8 hours. The pattern of the middle layer was cut using the laser cutter mentioned above. PDMS (10:1) was poured onto the PDMS-CNT/Au bilayer and cured at 80°C for 1 hour, forming a PDMS matrix layer. Next, the PDMS-CNT/Au electrode was peeled off from the glass substrate. The isolated ionic gel and PDMS-CNT/Au electrode were exposed to air plasma at 50 W for 20 s and gently stacked together, layer by layer, to integrate the pressure sensor array.

### Signal acquisition system

The MCU model (STM32H753IITC) has a working frequency up to 480 MHz. In the circuit, 15 ADG734BRUZ-REEL7 chips [4× SPDT (single pole double throw)] were used as the select multiplexers of row and column electrodes and the cross-talk compensation circuit switches. The CDC unit used a PCAP02AE capacitance-to-digital converter to read the capacitance change with a 19.3-bit resolution. The maximum scan rate of the signal acquisition system is 2000 cells s^−1^, and the current system sampling rate is 2.55 frames s^−1^ for the 28 × 28 sensor array. The data were first acquired by the MCU and then uploaded to a personal computer through a universal serial bus (USB) cable for real-time visual display and subsequent data processing.

### Morphology and composition characterization

The morphology of the iontronic skin, the electrode, and the IMIGs was characterized using cold-field SEM (Hitachi, SU8230). X-ray photoelectron spectroscopy data were collected using an x-ray photoelectron spectrometer (Escalab Xi+, Thermo Scientific) equipped with an aluminum x-ray radiation source of dual gun (1486.6 eV) with a 0.85-eV line width.

### Characterization of electrical properties of the sensor and the electrode

Electrochemical impedance spectroscopy of the sensor with an IMIG (loaded with 50 kPa) was tested using an electrochemical workstation (PGSTAT 302 N, Metrohm Autolab), and the testing frequency was ranged from 0.1 Hz to 1 MHz. The capacitance was measured using an locus control region (LCR) meter (E4980AL, KEYSIGHT) under 1 kHz and 1 V. The external pressure was applied and measured using a force gauge with a computer-controlled stage (XLD-20E, Jingkong Mechanical Testing Inc.). A sensor with a diameter of 5 mm was used to test the sensitivity and stability of the sensor under cyclic loading-unloading (compression, friction, and tension). The initial capacitance of the sensor was determined to be 3.0 pF; *C*_EDL1_ was very close to the initial capacitance and also determined to be 3.0 pF, and *C*_EDL2_ was determined to be 560 nF. The response time was measured using an LCR meter (TH2840B, Tonghui Inc.) under 10 kHz that has a higher sampling frequency. The resistances of the PDMS-CNT/Au electrode under bending and stretching were measured using a smart stretching tester (WS150-100) together with a Keithley 2400 multimeter.

### Characterization of mechanical properties

The PVA:H_3_PO_4_ gel, the PDMS-CNT composite, the PDMS-CNT/Au electrode, and pure PDMS were cut into dumbbell-shaped specimen to test their stress-stretch curves. The force was determined by the force gauge mentioned above, and a strain rate of 50 mm min^−1^ was applied during stretching. For the peeling test, a stiff polyester layer (50 μm in thickness) serving as a backing layer was adhered to the peeling pairs (with a thickness of 200 μm, plasma-treated) using silicon rubber adhesive (Sil-Poxy, Smooth-On Inc.). The width of the samples varied from 1.0 to 2.5 cm with a constant peeling speed of 50 mm min^−1^, and the interfacial toughness was calculated by eq. S1 in fig. S9.

### Integration of the skin to the prosthetic hand

Two types of sensor array (the iontronic skin with IMIGs and a control sample without IMIGs and embedded configuration; see fig. S8, A and B) were attached to a prosthetic hand (Bebionic Hand, Ottobock Inc.) using a polyethylene terephthalat–based double-sided tape of 6 μm in thickness. The capacitance signal of the sensing elements during prosthetic manipulation was recorded using a signal acquisition system for pressure mapping or using an LCR meter at a testing frequency of 1 kHz for the detection of sensing signal of a single pixel.

### Object recognition based on machine learning

The 1D-CNN, mainly including convolution and max pooling, was used to for grasping object recognition. For each object, 300 sets of data were collected, of which 220 sets are for training, 40 sets are for validation, and 40 sets are for testing. There were 1800 features for each object as the input of the 1D-CNN model, and the methods of convolution and max pooling were used to extract and amplify the features of the data. For the kernel of the convolution layer, the size and stride were 4 and 1, respectively. For the pool of max-pooling layer, the size and stride were 4 and 2, respectively. The discrete graphic card used for machine learning was TUF-RTX3090-O24G. All the operations were done by PyTorch.

## Supplementary Materials

### This PDF file includes:

Figs. S1 to S19 Legends for movies S1 to S3 References
