# Supplementary material for: Embedment of sensing elements for robust, highly sensitive, and cross-talk–free iontronic skins for robotics applications
Source: Sci Adv. 2023 Mar 3;9(9):eadf8831. doi: 10.1126/sciadv.adf8831 (PMC9984179; doi:10.1126/sciadv.adf8831)
Supplement: Supplementary file 1 — Figs. S1 to S19 Legends for movies S1 to S3 References [file sciadv.adf8831_sm.pdf]

Science Advances
AAAS

Supplementary Materials for

Embedment of sensing elements for robust, highly sensitive, and cross-talk–free iontronic skins for robotics applications

Junli Shi *et al.*

Corresponding author: Chuan Fei Guo, guocf@sustech.edu.cn

Sci. Adv. **9**, eadf8831 (2023)
DOI: 10.1126/sciadv.adf8831

The PDF file includes:

Figs. S1 to S19
Legends for movies S1 to S3
References

Other Supplementary Material for this manuscript includes the following:

Movies S1 to S3

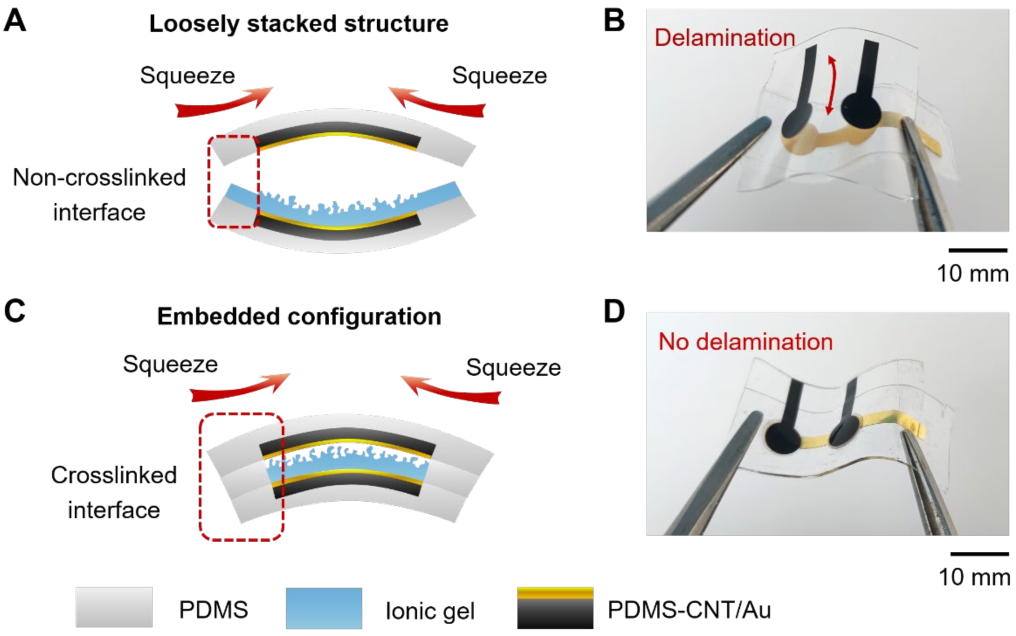

Fig. S1. Mechanical stability of iontronic sensors with a loosely stacked structure and with embedded IMIGs. (A) Schematic of the sensor with a loosely stacked structure. **(B)** Delamination occurs when squeezing the sensor. **(C)** Schematic of the sensor that embed the IMIG in elastomeric matrix with a fully integrated structure. **(D)** Optical image of the sensor with the IMIG, showing no delamination upon squeezing.

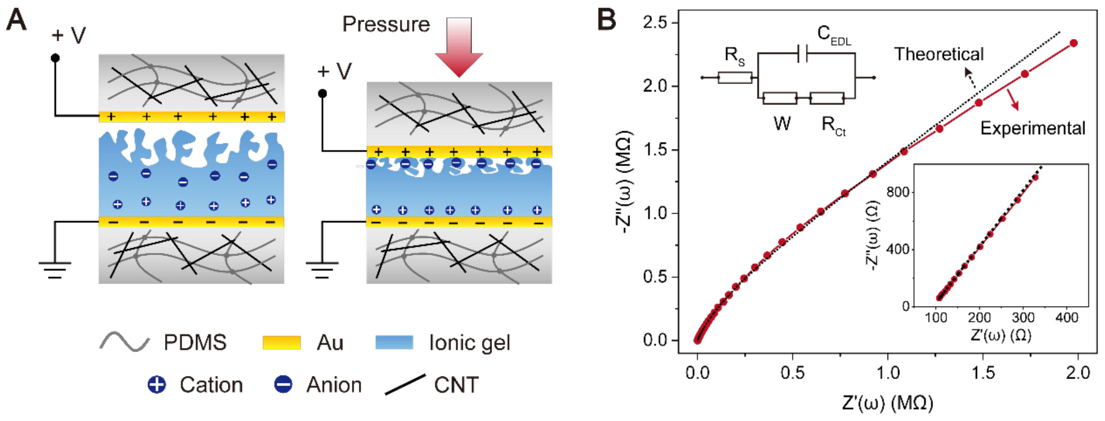

Fig. S2. Sensing mechanism of the iontronic sensor and its electrochemical impedance spectrum. (A) Sensing mechanism of the sensor. The capacitive signal is determined by the iontronic interfaces. **(B)** Nyquist plot of the complex impedance measured under a load of 50 kPa. The inset shows the equivalent circuit of the sensor. Here R_S represents the internal resistance of the electrolyte, R_{Ct} is the charge transfer resistance at the electrode/electrolyte interface and W is the Warburg impedance. The fitted curve and the equivalent circuit indicate that the EDLs are formed at the electrode-ionic gel interface. It is noted that the PDMS-CNT/Au electrode exhibits non-porous properties. Meanwhile, the ionic gel (PVA:H_3PO_4) that contacts with the electrodes has only a single phase. Thus, the rapid ion diffusion results in that the measured Nyquist plot having a parabolic shape (*34*).

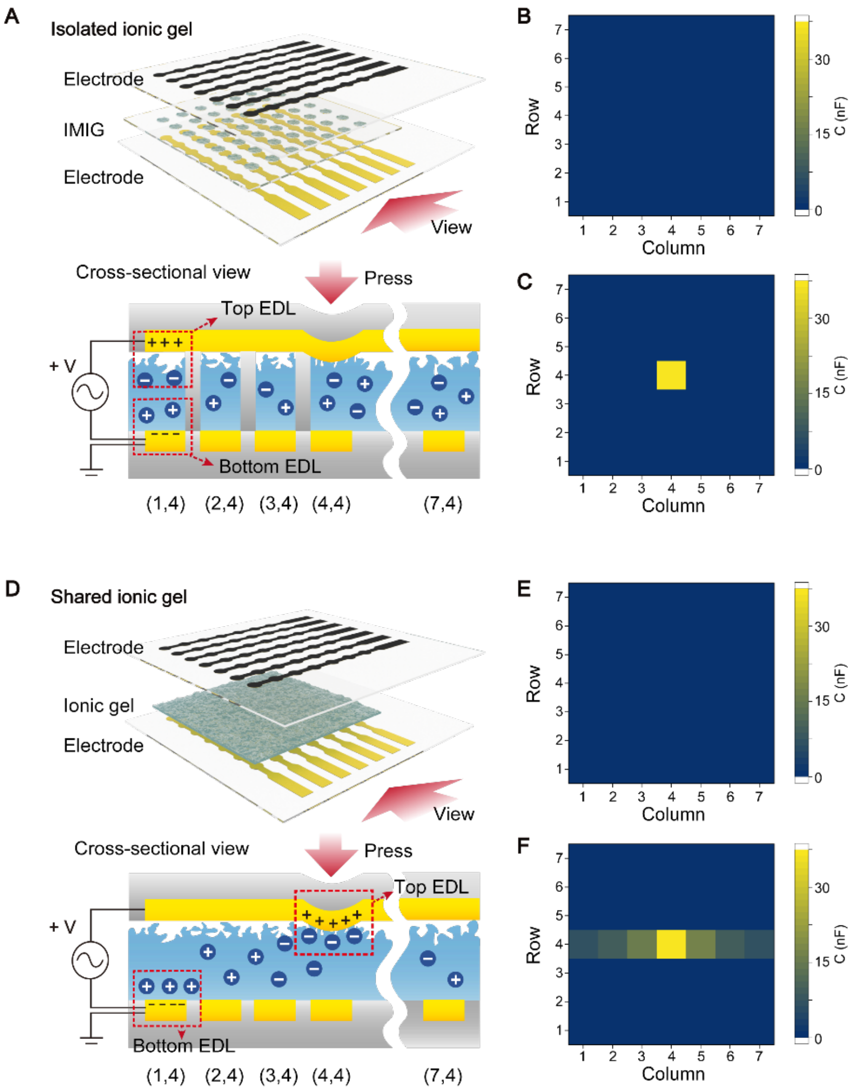

Fig. S3. Origin of crosstalk between sensors of the same row (or column) in a sensor array for the cases of (A-C) using isolated ionic layers in different sensing elements and (D-F) sharing an iontronic layer. Assume that in both cases sensing element (4,4) is under pressure, and sensing element (1,4) is under measurement. (**A**) For the first case, the ionic gel for each sensing element is isolated, and thus ion migration is confined to a single sensing element. Because sensing element (4,4) is not connected in the circuit, the measured capacitance can be considered as the bottom EDL and the top EDL of sensing element (1,4) in series. (**B**,**C**) Experimental data show that when pressing on the pixel (4,4), no signal from other pixels was detected. (**D**) For the second case (using shared ionic gel), when a pressure is applied to sensing element (4,4), a bottom EDL of sensing element (1,4) and a top EDL of sensing element (4,4), which increases with pressure, are connected in series, and thus there is a large capacitance signal of sensing element (1,4), leading to crosstalk. **(E,F)** When pressing on the pixel (4,4), crosstalk (up to 39%) of the pixels in the same row can be observed experimentally.

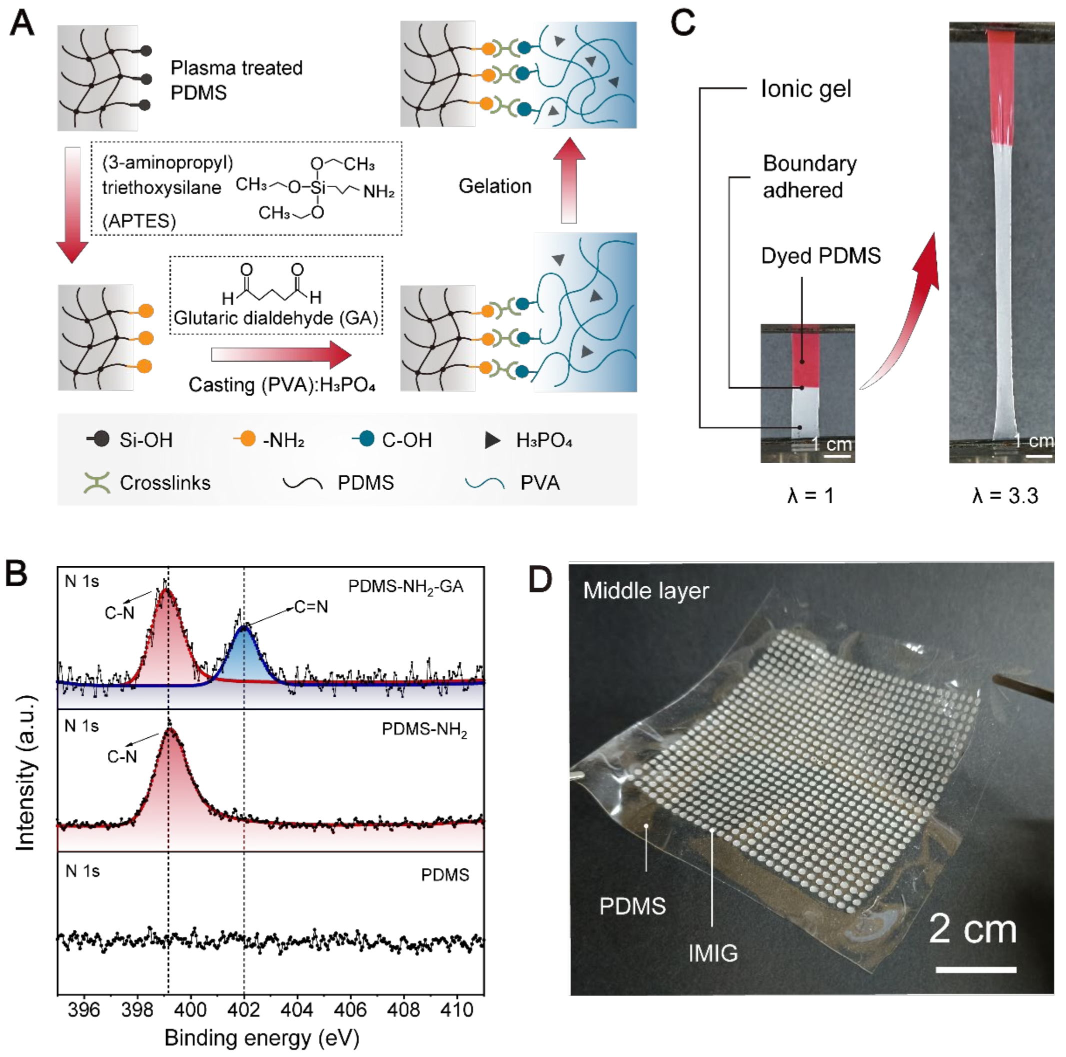

Fig. S4. Crosslinking between the PDMS matrix (the middle layer) and the IMIGs. (A) Schematic of the crosslinked interface between the perforated PDMS and the ionic gel. **(B)** Compositional analysis by detecting the N 1s peaks using X-ray photoelectron spectroscopy. No N 1s peak is detected for untreated PDMS, while a peak with a binding energy centered at 399.3 eV corresponding to the C-N bond (*42*) is detected when the PDMS is grafted with (3-aminopropyl) triethoxysilane (APTES). After glutaric dialdehyde (GA) treatment, a doublet corresponding to the C=N bond (*44*) (401.9 eV) and the C-N bond (399.3 eV), of which the former is induced in the Schiff base reaction, can be detected. **(C)** Elongation of a crosslinked PDMS-IMIG pair. Here PDMS is dyed. The crosslinked PDMS-IMIG pair can be elongated 3.3 times the initial length. **(D)** Photograph of the middle layer with the IMIGs embedded in the hole array of the perforated PDMS.

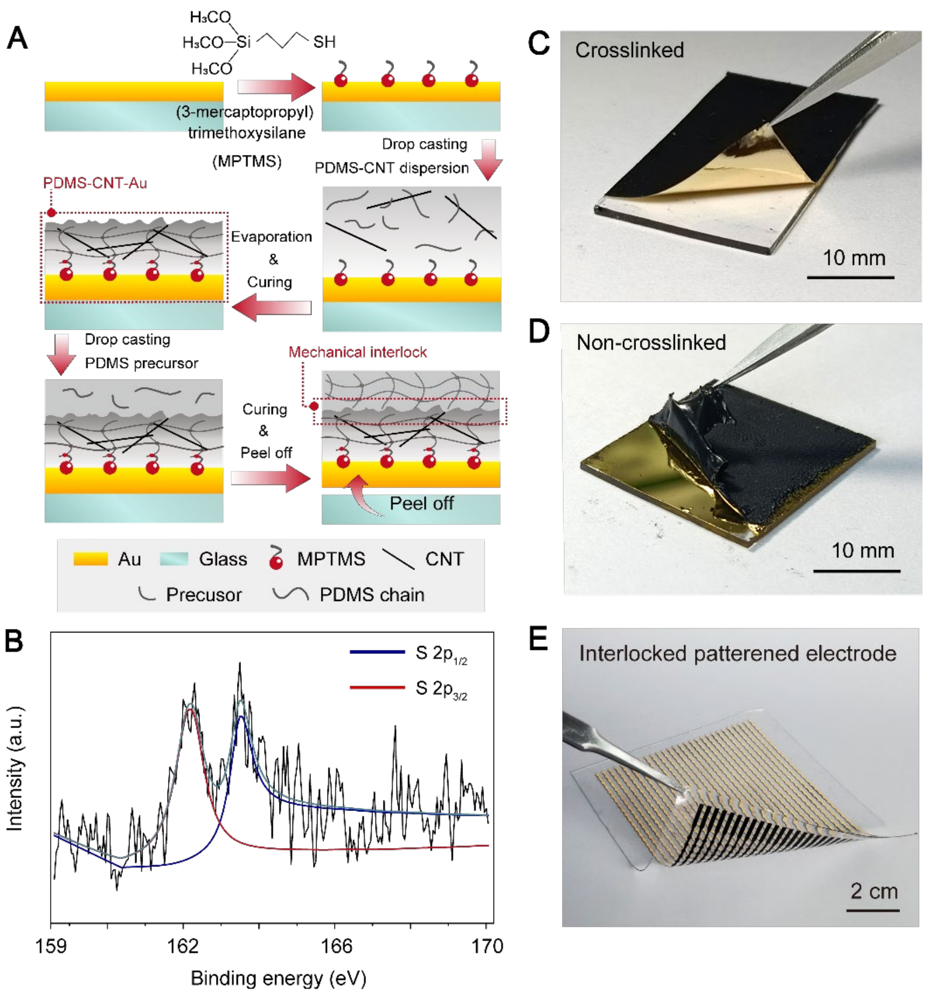

Fig. S5. Fabrication and characterization of the PDMS-CNT/Au electrode. (A) Schematic for the fabrication of the PDMS-CNT/Au bilayer. The surface of the Au film is pre-modified by introducing a (3-mercaptopropyl)trimethoxysilane (MPTMS) self-assembled monolayer, which forms a strong Au-S bond with the Au film. On the other hand, the flat surface of the PDMS-CNT composite is grafted with the Si-OCH_3 groups. A covalent bond is formed when the modified surfaces of the Au film and the PDMS-CNT composite are in contact (*44*). Subsequently, PDMS precursor is casted on the surface of the PDMS-CNT composite to encapsulate the cured PDMS-CNT/Au electrode. The cured PDMS strongly adheres to the PDMS-CNT composite through mechanical interlock. **(B)** S 2p XPS spectra of MPTMS on Au surface. The S 2p signal indicates that the MPTMS silane is successfully grafted on Au surface (*45*). **(C)** Photograph showing that the Au film treated by MPTMS can be fully transferred to the PDMS-CNT layer. **(D)** Photograph showing that non-treated Au film cannot be transferred to PDMS-CNT. **(E)** Photograph of a layer of patterned electrode, for which the PDMS-CNT/Au bilayer is interlocked with the PDMS matrix, serving as the patterned electrode of the iontronic skin in this study. The width of the connecting wires is 0.8 mm.

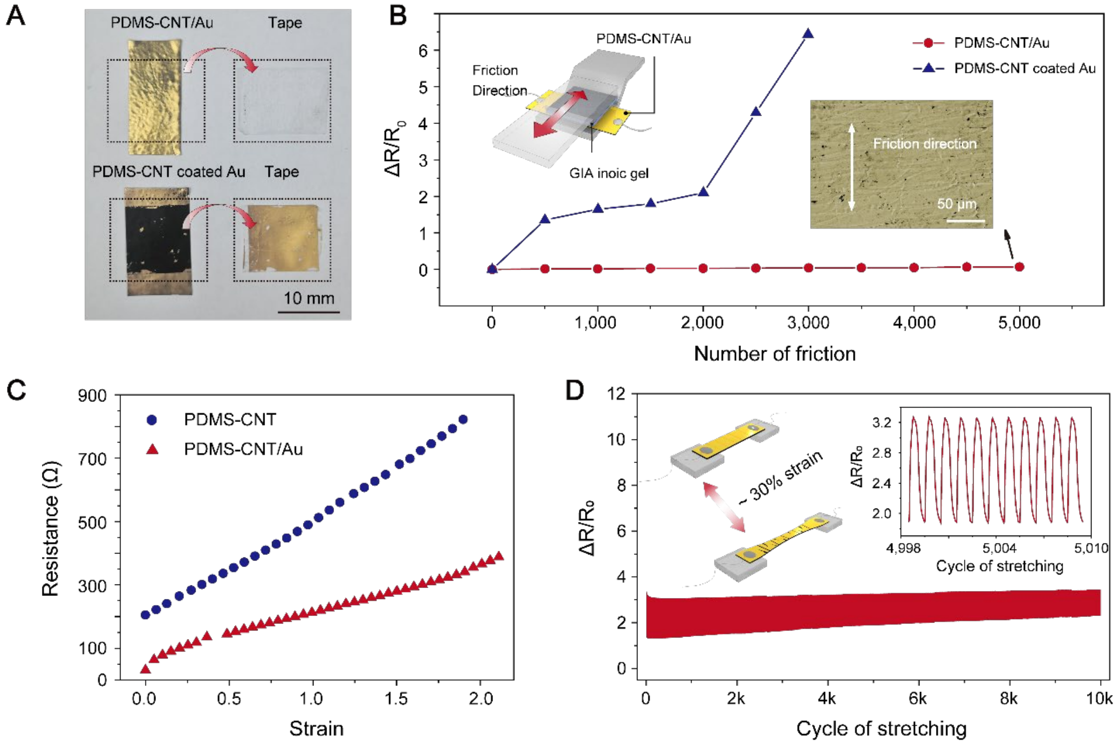

Fig. S6. Electrical properties of the PDMS-CNT/Au electrode. (A) Tape peeling test of two types of electrodes. The top image shows the PDMS-CNT/Au electrode used in this work, and the Au film cannot be peeled off using a piece of 3M tape. The bottom one shows that a layer of 100 nm Au film directly deposited on PDMS-CNT surface without crosslinking can be stripped away using 3M tape due to the poor adhesion between Au and the PDMS-CNT composite. **(B)** Normalized change in resistance of the PDMS-CNT/Au electrode (circles) and a control sample for which the Au film is not crosslinked (triangles). The insets show the setup for the rubbing test, and an SEM image of the surface of the PDMS-CNT/Au electrode after 5,000 rubbing cycles (no damage of the Au film is seen). **(C)** Resistance as a function of strain for the PDMS-CNT layer and the PDMS-CNT/Au electrode, showing that the latter has a smaller resistance than the former. **(D)** Change in resistance of the PDMS-CNT/Au electrode during 10k stretch (30%)-release cycles. The first cycle induces a relatively large change in resistance, which may stem from the crack formation of the Au film and the realignment of CNTs (*46*).

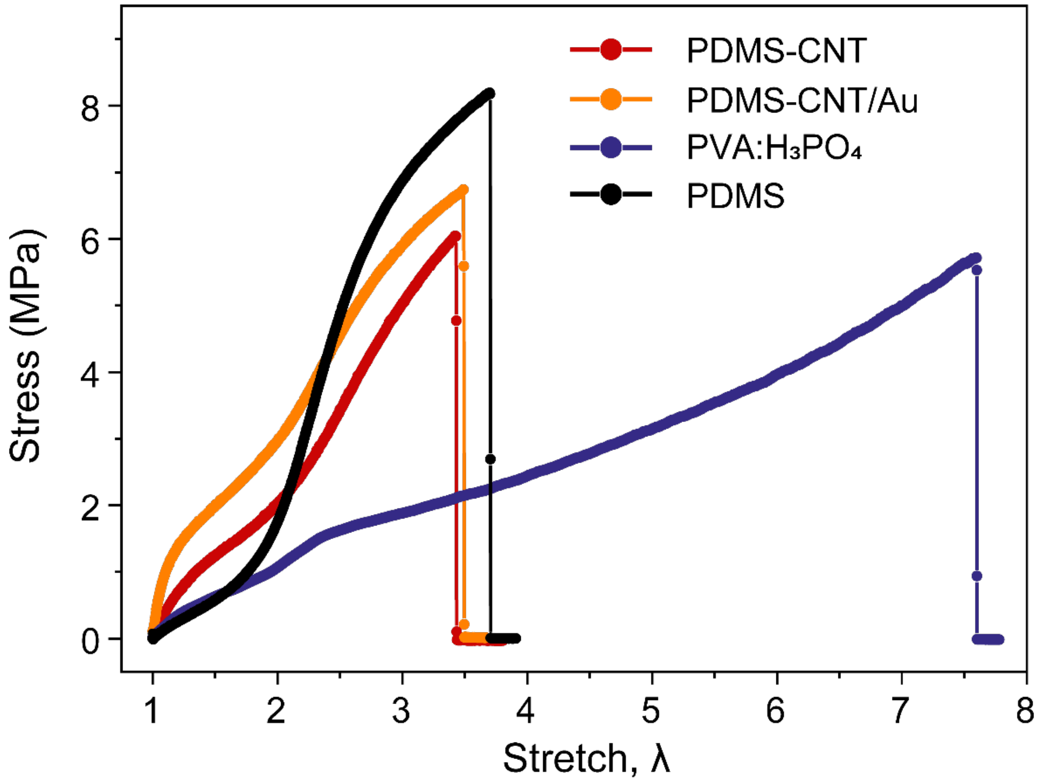

Fig. S7. Stress-stretch curves of pure PDMS, PVA:H_3PO_4, the PDMS-CNT composite, and the PDMS-CNT/Au electrode. The PVA:H_3PO_4 ionic gel (1.3 MPa) has a close Young's modulus with PDMS (1.6 MPa).

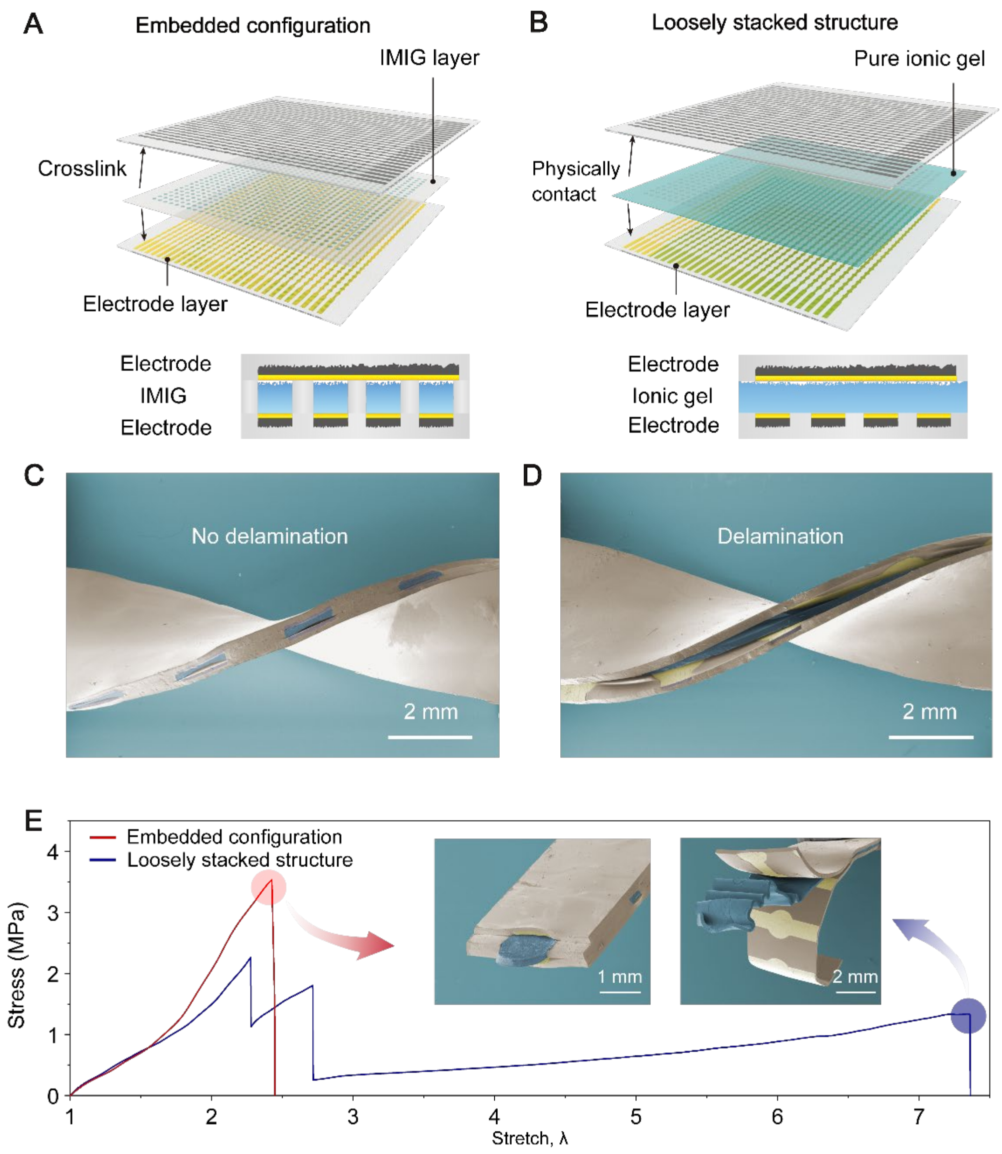

Fig. S8. Structure and mechanical stability of sensor arrays with the embedded configuration and with a loosely stacked configuration. (A) Structure of the sensor array with the embedded configuration. **(B)** Sensor array with a conventional structure, for which the interlayers are non-bonded. **(C)** and **(D),** SEM images showing the cross section of the two sensor arrays under twisting. No delamination is seen for the embedded configuration. **(E)** Stress-strain curves of the two sensor arrays. Insets show corresponding SEM images of ruptured samples. Our sensor array with the embedded configuration shows no delamination, and corresponding stress-stretch curve is similar to that of a pure material. By contrast, the stress-stretch curve of the loosely stacked sample has a jagged shape due to the layer-by-layer rupture, which is verified in the SEM observation.

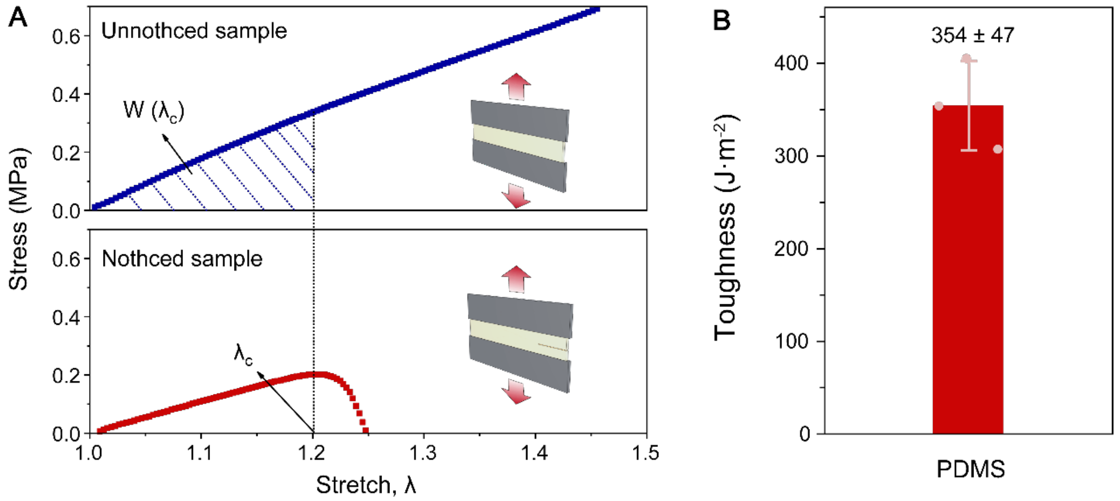

Fig. S9. Intrinsic toughness of PDMS. (A) Stress-stretch curves of samples with and without a notch. **(B)** Interfacial toughness of PDMS. The interfacial toughness Γ (J·m^{-2}) is determined by the equations below (*47, 48*):

$$\Gamma = \frac{F}{d} \cdot (1 - \cos\theta) \tag{1}$$

$$\Gamma = \Gamma_0 + \Gamma_D \tag{2}$$

where F is the applied peeling force; d is the width of the sample; and θ is the angle between the peeling pair, which is 180° in this study. Γ_0 represents the intrinsic work of adhesion energy per unit area, and Γ_D is the dissipated energy per unit area caused by structure deformation.

The intrinsic toughness (Γ_{in}) of a material is defined as the energy per unit area dissipated during crack formation, which is often measured by tearing (*49*).

For the peeling test, the raw materials used in the iontronic skin were cut into a rectangular shape with a length of 60 mm, a height of 10 mm, and a thickness of 0.1 mm. The samples were adhered between two polymethyl methacrylate grippers. Samples for each material were divided into two different types—one without notch, and the other with a notch of 20 mm long, as shown in **fig. S9A**. For the unnotched sample, the area beneath the stress-stretch curve represents the elastic energy density (W_λ) of the material. For the notched sample, the stretch at which the crack start to extend is defined as λ_c, and the toughness can be expressed as:

$$\Gamma_{in} = W_{\lambda_c} \cdot H \tag{3}$$

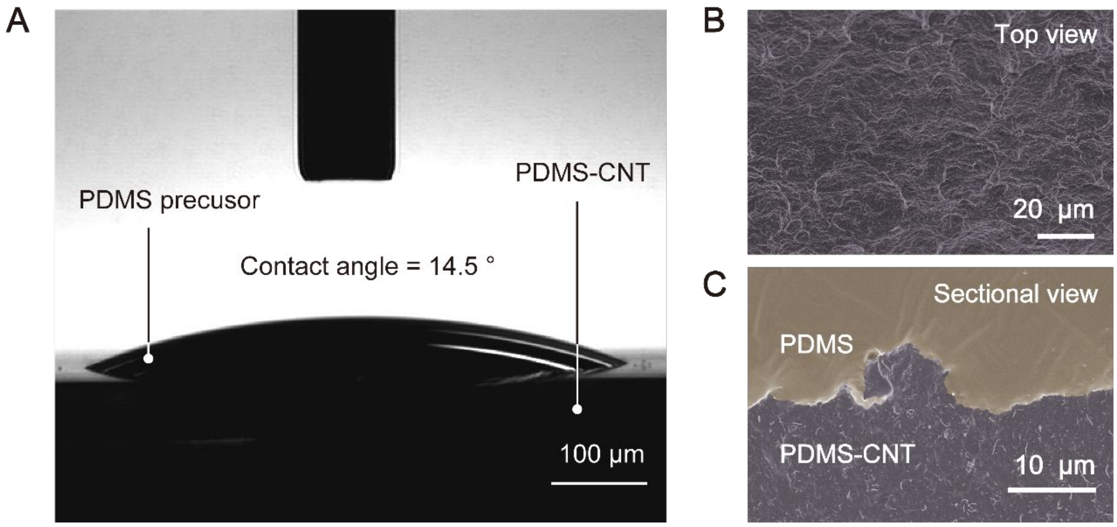

Fig. S10. Interlocked interface between PDMS-CNT composite and PDMS matrix. **(A)** Contact angle between the PDMS precursor (liquid) and the PDMS-CNT composite (solid), showing that PDMS precursor can well wet the PDMS-CNT composite that has a rough surface. **(B)** SEM image showing the rough surface of the PDMS-CNT composite. **(C)** Cross-sectional view SEM image of the interface between the PDMS-CNT composite and the PDMS encapsulation layer.

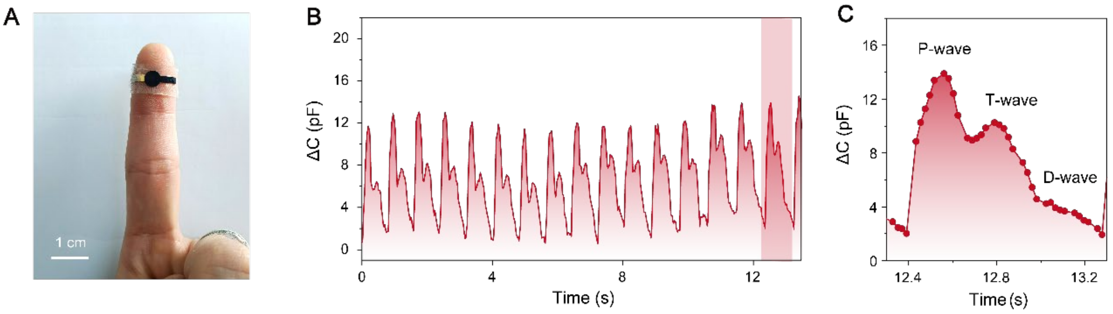

Fig. S11. Fingertip pulse detection using our sensor. (A) Photograph of the sensor wrapped on a fingertip**.** The sensor can well comply with the skin due to its intrinsic softness. **(B)** and **(C)** Pulse wave detected using the sensor, showing clear P-, T-, and D-waves (*50*).

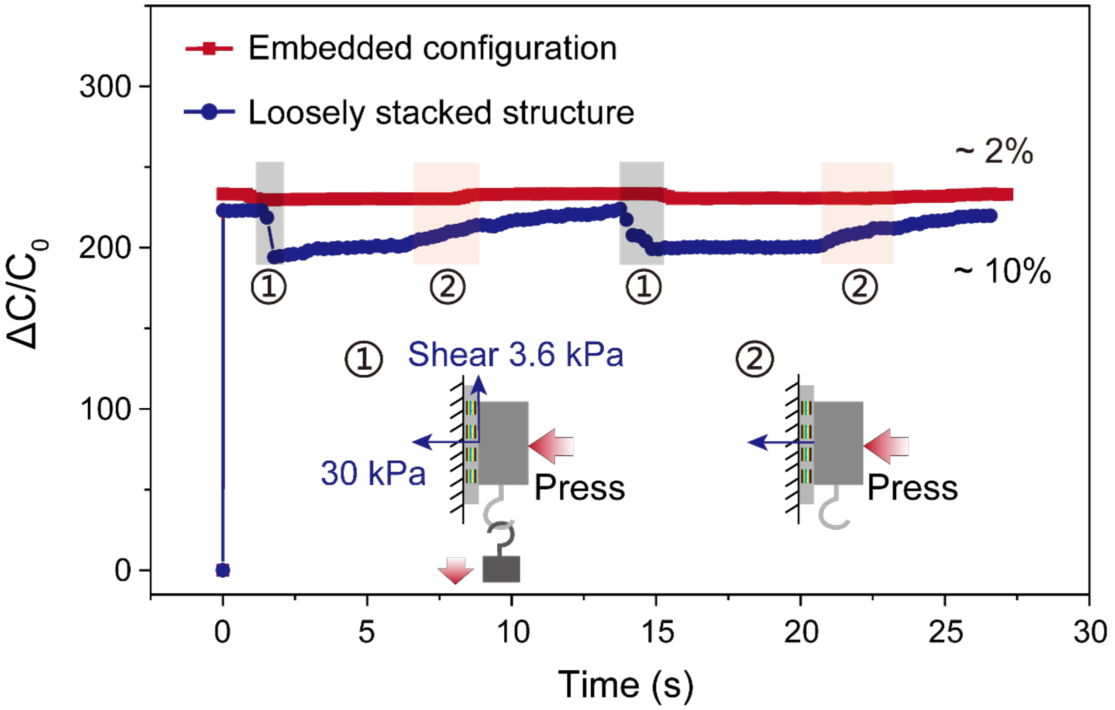

Fig. S12. Effect of static shear stress on sensing signal for sensors with and without the embedded configuration. Inset shows the setup of the test. The inter-hole walls in the middle layer limit the lateral displacement between the electrode and the ionic material, resulting in poor response to shear stress.

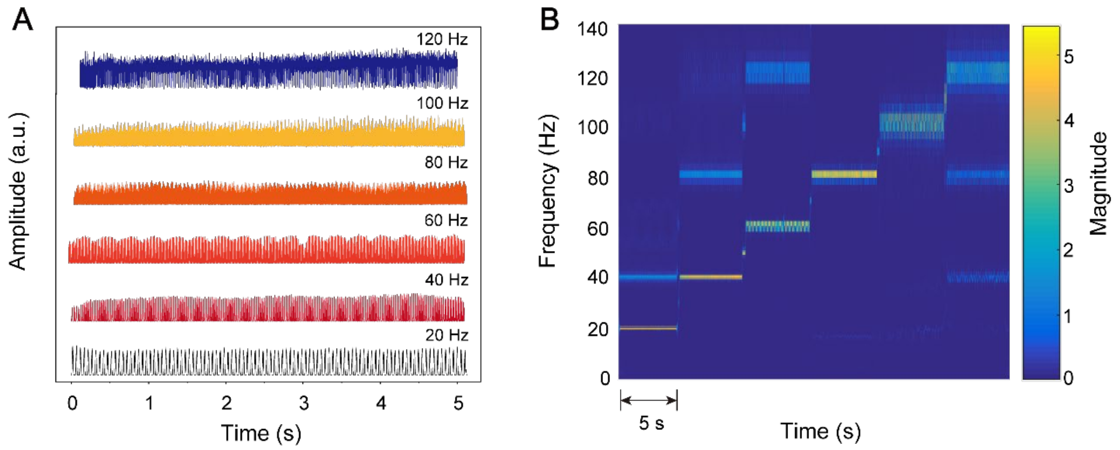

Fig. S13. Response of the sensor under different vibrational frequencies. (A) Capacitance change when loading vibrations with different frequencies. **(B)** Continuous wavelet transform of the signals collected in panel **(A)** showing that the sensor can detect frequencies of at least 120 Hz.

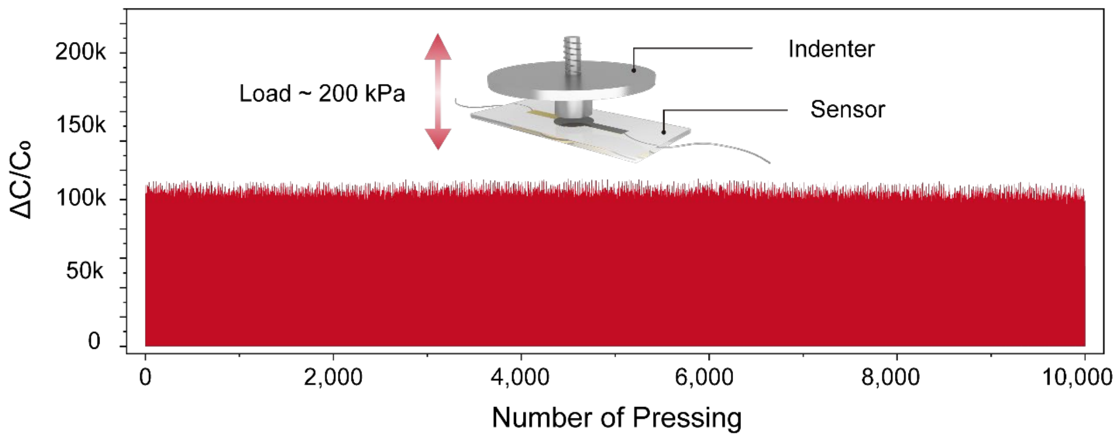

Fig. S14. Response of the sensor under cyclic loading (200 kPa) and release over 10,000 times. The result indicates that our embedded sensor has excellent stability over 10k loading-release cycles.

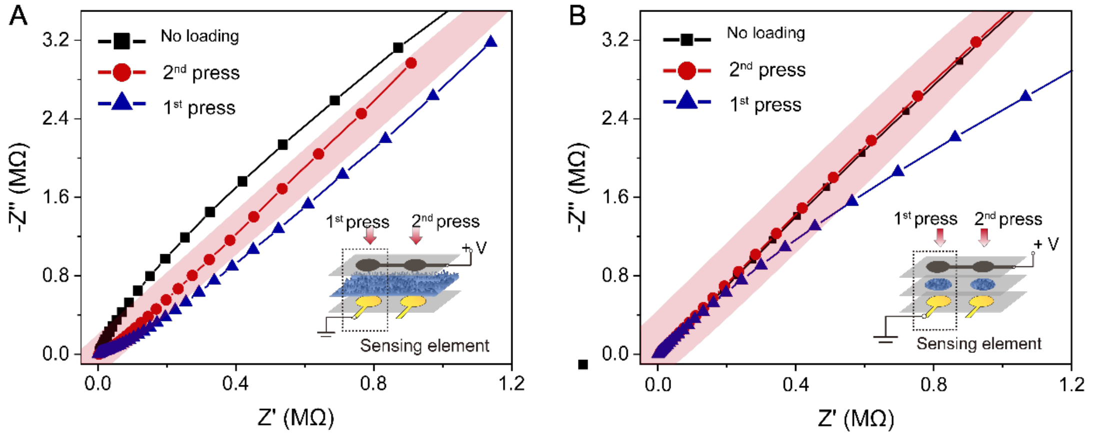

Fig. S15. Nyquist plots of the two sensor configurations. (A) Sensing elements with shared ionic gel. **(B)** Sensing elements with IMIGs as the ionic material. For the case using the IMIGs, no change in signal of a target sensing element occurs when touching the neighboring sensing element because of no ion migration.

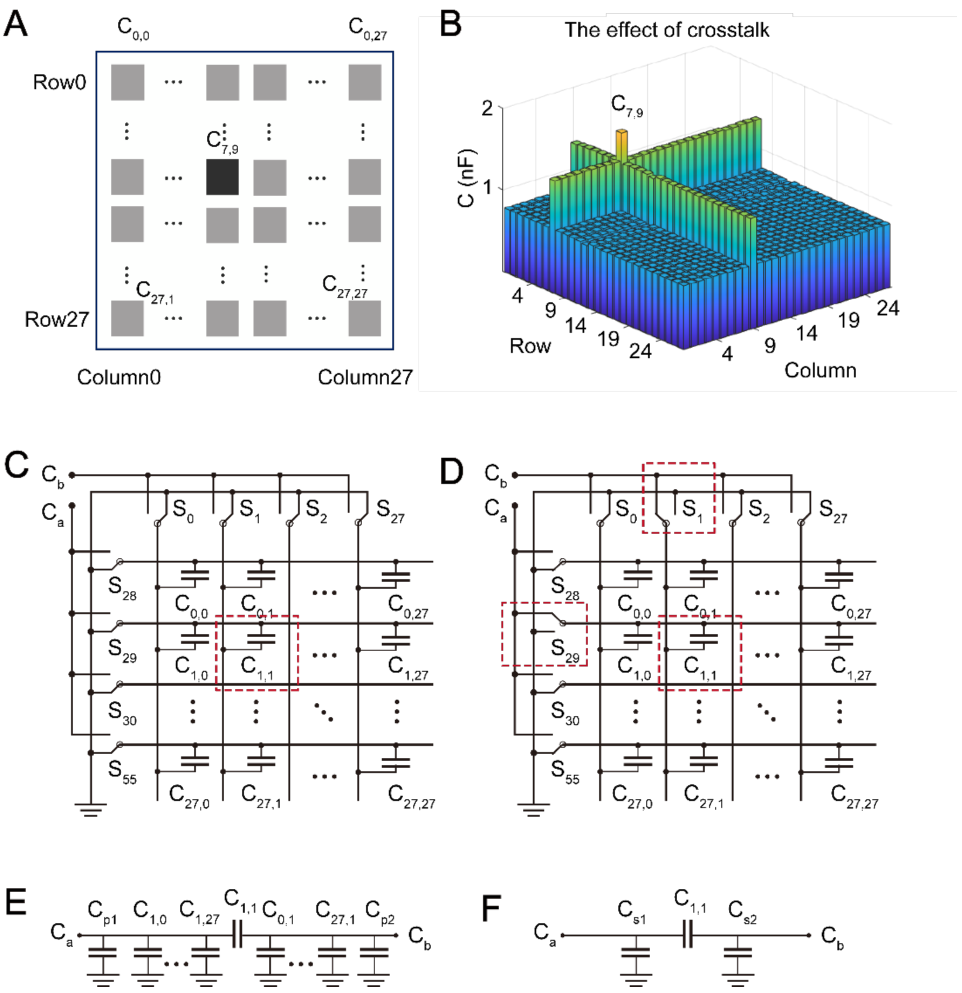

Fig. S16. Origin of crosstalk and electrical equivalent diagram of the sensor array. **(A)** Distributed view of the sensor array (28×28 pixels). When applying a pressure to a randomly selected capacitor $C_{7,9}$, the capacitance distribution of the sensor array collected by the readout circuit exhibiting giant crosstalk for the sensors in the same row/column, as shown in panel (**B**). **(C)** Circuit diagram of the sensor array and corresponding analog switches. We take C_a and C_b as circuit nodes to analyze the crosstalk problem. Taking capacitor $C_{1,1}$ for example, the analog switch of $C_{1,1}$ is connected to C_a and C_b through S1 and S29, respectively; and other switches are connected to the GND line so that other capacitors will not affect sensor $C_{1,1}$, as shown in panel (**D**). **(E)** A simplified circuit of the case of panel (**D**). $C_{1,1}$ is connected in series with C_a and C_b. Parasitic capacitors C_{p1} and C_{p2} are considered in the circuit. **(F)** A further simplified circuit, for which the parallel capacitors (including the parasitic capacitors) in the same column and the same row are regarded as a single capacitor C_{s1} and C_{s2}, respectively. The error in reading the capacitance of the target capacitor $C_{1,1}$ comes from C_{s1} and C_{s2}.

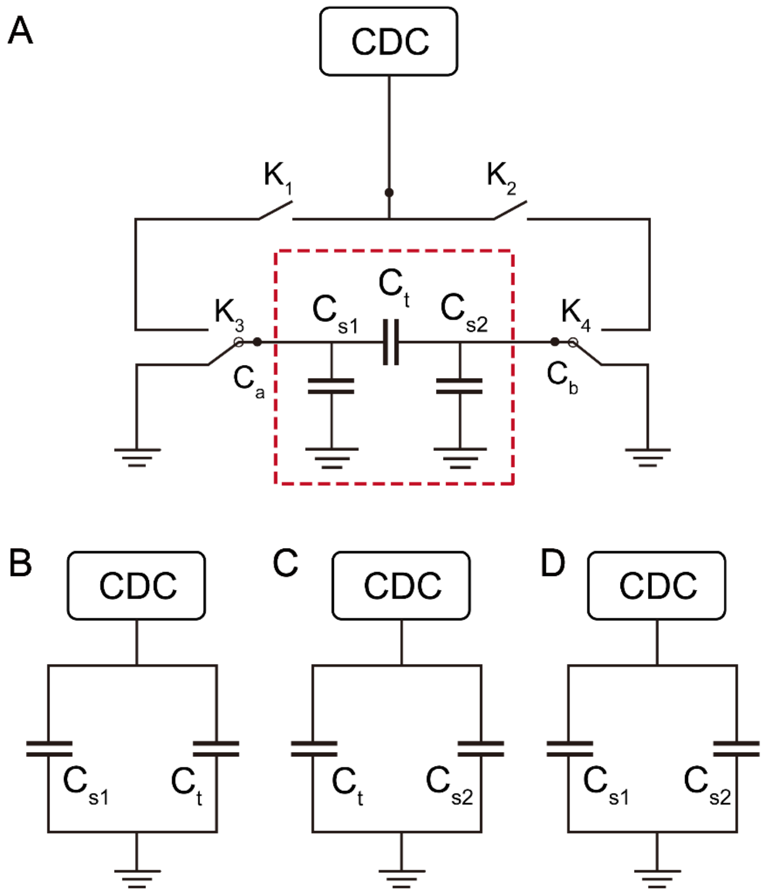

Fig. S17. Diagram of crosstalk compensation circuit and equivalent circuit for each step. (A) Connection mode of the crosstalk compensation circuit consists of four analog switches. **(B)** Step 1: K_1 is closed, and K_2 is disconnected; K_3 is connected to K_1, and K_4 is connected to the GND line. At this step, both electrodes of C_{s2} are connected to the GND line, and C_{s1} and C_t are in parallel. **(C)** Step 2: K_2 is closed, and K_1 is disconnected; K_4 is connected to K_2, and K_3 is connected to the GND line. At this step, both electrodes of C_{s1} are connected to the GND line, and C_t and C_{s2} are in parallel. **(D)** Step 3: Both K_1 and K_2 are closed; K_3 is connected to K_1; K_4 is connected to K2; C_t is shorted, and C_{s1} and C_{s2} are in parallel.

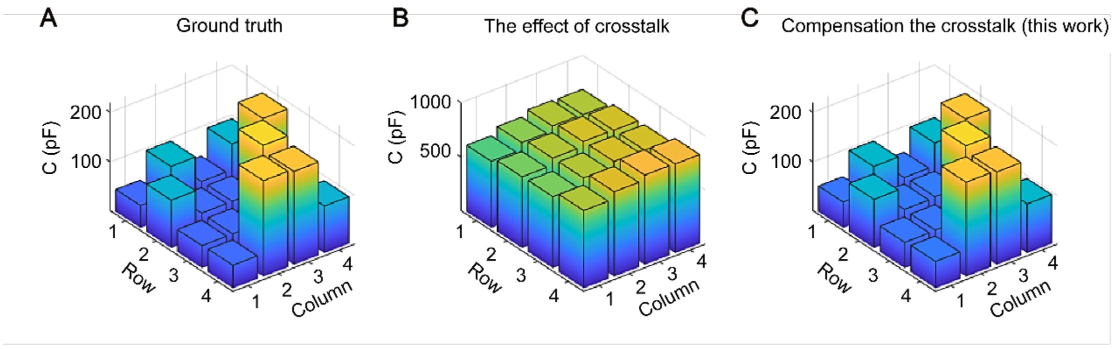

Fig. S18. Signal acquisition diagram of a 4×4 fixed capacitor array. (A) Capacitance distribution of a 4×4 capacitor array as the ground truth measured using an LCR meter. The capacitance of each capacitor is randomly distributed. **(B)** Capacitance distribution of the array when it is connected to the signal acquisition system but without connecting to the crosstalk compensation module, showing that the capacitance distribution is different from that measured using the LCR meter. **(C)** When connected to the crosstalk compensation module, the capacitance distribution of the signal array after crosstalk compensation is quite close to that measured using the LCR meter.

The crosstalk of the target capacitor stems from other capacitors in the same row or column. It is equivalent to two grounding capacitors in parallel with the upper and lower electrodes of the target capacitor. Therefore, a compensation module is designed to eliminate the crosstalk between the capacitors of the same row or column. The specific steps of the design are as follows:

Step 1: Switch K_1 is connected to the CDC unit; K_2 is turned off; K_3 is connected to K_1, and K_4 is grounded. Next, current circuit capacitance C_1 is read through the CDC unit. Capacitance C_1 is expressed as:

$$C_1=C_t+C_{s1} \tag{4}$$

where C_t is the target capacitance, and C_{s1} is the parallel crosstalk capacitance of the same row.

Step 2: Switch K_2 is connected to the CDC unit, K_1 is turned off, K_4 is connected to K_2, and K_3 is grounded. Current circuit capacitance C_2 is then readout through the CDC unit. Capacitance C_2 is expressed as:

$$C_2=C_t+C_{s2} \tag{5}$$

where C_{s2} is the parallel crosstalk capacitance of the same column.

Step 3: Both K_1 and K_2 are connected to the CDC unit. K_3 is connected to K_1, and K_4 is connected to K_2. Current circuit capacitance C_3 through CDC unit is read:

$$C_3=C_{s1}+C_{s2} \tag{6}$$

After sending the values of C_1, C_2, and C_3 to the MCU, the C_t value after decoupling the crosstalk is determined as follows:

$$C_t=(C_1+C_2-C_3)/2 \tag{7}$$

The crosstalk of the target capacitance can thus be eliminated, and then each sensing element in the array is switched in turn, row by column. Finally, the capacitance of each sensing element is calculated. All data are combined into a complete array data

frame and uploaded to a PC.

We verified the performance of the signal acquisition system from two aspects: accuracy, and crosstalk compensation ability. A fixed capacitor array (4×4) was designed to verify the effect of the acquisition board, as shown in **fig. S18.** We used an LCR meter to separately test the capacitance values of each capacitor as the ground truth (**fig. S18A**). Before the crosstalk compensation module is used, giant crosstalk between sensing elements is observed (**fig. S18B**). By contrast, after using the crosstalk compensation module, the crosstalk can be well decoupled, as shown in **fig. S18C**. The root mean square error (*RMSE*) is used to characterize the accuracy compared with the ground truth:

$$RMSE = \sqrt{\frac{\sum_{i=1}^{n}(\hat{C}_i - C_i)^2}{n}} \tag{8}$$

where $\hat{C}_i$ is the detected capacitance value, and C_i is the ground truth. According to Eq. (8), the *RMSE* of the case without crosstalk compensation is 632.3, while the *RMSE* after using the crosstalk compensation module decreases to only 5.3.

Furthermore, crosstalk rate is used as an index to evaluate the decoupling effect during crosstalk compensation. The crosstalk rate is defined as the ratio of the change in capacitance of the capacitor in the same row or column to the capacitance change of the capacitor itself when a pressure is applied.

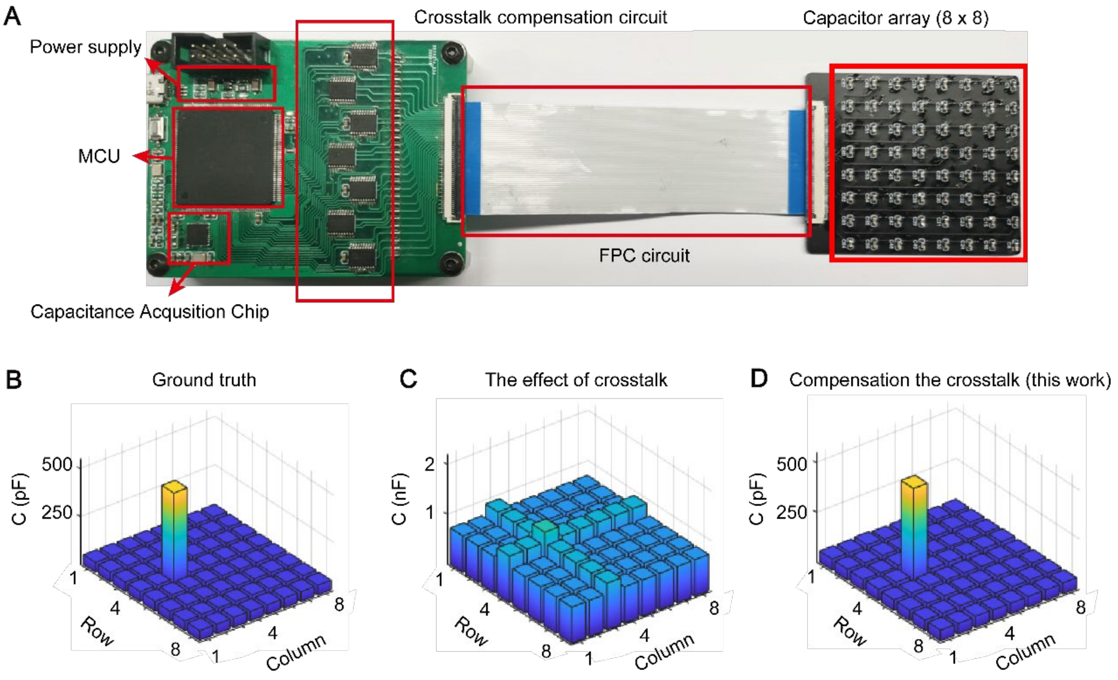

Fig. 19. Crosstalk compensation of an 8×8 fixed capacitor array. (A) Photograph of the readout circuit connecting to the capacitor array (8×8). **(B)** Capacitance distribution of the fixed capacitor array as the ground truth measured using an LCR meter, showing that capacitance values of the capacitors are uniformly distributed except for a larger capacitor that is used to mimic a sensor under pressure. **(C)** When the fixed capacitor array is connected to the signal acquisition system but without connecting to the crosstalk compensation module, crosstalk is observed between the larger capacitor and other capacitors in the same row/column. **(D)** When connected the crosstalk compensation module, crosstalk is effectively suppressed, and the result is very close to that measured using the LCR meter.

Taking a fixed capacitor array (8×8) for example. We first replace a capacitor with a large fixed capacitor to simulate the capacitance change of sensors under pressure. Next, real capacitance distributions is shown in **fig. S19B**. When the crosstalk compensation module is not used, the crosstalk rate of each sensing element is greater than 75% (**fig. S19C**). Therefore, it is difficult to precisely record the capacitance values of the elements in the array. By contrast, when the crosstalk compensation module is used to eliminate crosstalk, the crosstalk rate of each cell reduces to only 0.67% (**fig. S19D**).

Movie S1: **Peeling process of the iontronic skin with embedded configuration.**

Movie S2: **Tactile demonstration.**

Movie S3: **Real-time object recognition.**

REFERENCES AND NOTES

1. Y.-C. Huang, Y. Liu, C. Ma, H. C. Cheng, Q. He, H. Wu, C. Wang, C.Y. Lin, Y. Huang, X. Duan, Sensitive pressure sensors based on conductive microstructured air-gap gates and two-dimensional semiconductor transistors. *Nat. Electron.* **3**, 59–69 (2020).

2. G. Gu, N. Zhang, H. Xu, S. Lin, Y. Yu, G. Chai, L. Ge, H. Yang, Q. Shao, X. Sheng, X. Zhu, X. Zhao, A soft neuroprosthetic hand providing simultaneous myoelectric control and tactile feedback. *Nat. Biomed. Eng.* (2021).

3. P. Kieliba, D. Clode, R. O. Maimon-Mor, T. R. Makin, Robotic hand augmentation drives changes in neural body representation. *Sci. Robot.* **6**, eabd7935 (2021).

4. M. Wang, Y. Luo, T. Wang, C. Wan, L. Pan, S. Pan, K. He, A. Neo, X. Chen, Artificial skin perception. *Adv. Mater.* **33**, e2003014 (2021).

5. X. Yu, Z. Xie, Y. Yu, J. Lee, A. Vazquez-Guardado, H. Luan, J. Ruban, X. Ning, A. Akhtar, D. Li, B. Ji, Y. Liu, R. Sun, J. Cao, Q. Huo, Y. Zhong, C. M. Lee, S. Y. Kim, P. Gutruf, C. Zhang, Y. Xue, Q. Guo, A. Chempakasseril, P. Tian, W. Lu, J. Y. Jeong, Y. J. Yu, J. Cornman, C. S. Tan, B. H. Kim, K. H. Lee, X. Feng, Y. Huang, J. A. Rogers, Skin-integrated wireless haptic interfaces for virtual and augmented reality. *Nature* **575**, 473–479 (2019).

6. H. Kim, J. Choi, K. K. Kim, P. Won, S. Hong, S. H. Ko, Biomimetic chameleon soft robot with artificial crypsis and disruptive coloration skin. *Nat. Commun.* **12**, 4658 (2021).

7. Y. Zhao, S. Zhang, T. Yu, Y. Zhang, G. Ye, H. Cui, C. He, W. Jiang, Y. Zhai, C. Lu, X. Gu, N. Liu, Ultra-conformal skin electrodes with synergistically enhanced conductivity for long-time and low-motion artifact epidermal electrophysiology. *Nat. Commun.* **12**, 4880 (2021).

8. Z. Shen, Z. Zhang, N. Zhang, J. Li, P. Zhou, F. Hu, Y. Rong, B. Lu, G. Gu, High-stretchability, ultralow-hysteresis conducting polymer hydrogel strain sensors for soft machines. *Adv. Mater.* **34**, 2203650 (2022).

9. G. Gu, H. Xu, S. Peng, L. Li, S. Chen, T. Lu, X. Guo, Integrated soft ionotronic skin with stretchable and transparent hydrogel-elastomer ionic sensors for hand-motion monitoring. *Soft Robot.* **6**, 368–376 (2019).

10. J. H. Zhang, Z. Li, J. Xu, J. Li, K. Yan, W. Cheng, M. Xin, T. Zhu, J. Du, S. Chen, X. An, Z. Zhou, L. Cheng, S. Ying, J. Zhang, X. Gao, Q. Zhang, X. Jia, Y. Shi, L. Pan, Versatile self-assembled electrospun micropyramid arrays for high-performance on-skin devices with minimal sensory interference. *Nat. Commun.* **13**, 5839 (2022).

11. J.-H. Kim, C. Marcus, R. Ono, D. Sadat, A. Mirzazadeh, M. Jens, S. Fernandez, S. Zheng, T. Durak, C. Dagdeviren, A conformable sensory face mask for decoding biological and environmental signals. *Nat. Electron.* **5**, 794–807 (2022).

12. Z. Zhou, K. Chen, X. Li, S. Zhang, Y. Wu, Y. Zhou, K. Meng, C. Sun, Q. He, W. Fan, E. Fan, Z. Lin, X. Tan, W. Deng, J. Yang, J. Chen, Sign-to-speech translation using machine-learning-assisted stretchable sensor arrays. *Nat. Electron.* **3**, 571–578 (2020).

13. N. Bai, L. Wang, Q. Wang, J. Deng, Y. Wang, P. Lu, J. Huang, G. Li, Y. Zhang, J. Yang, K. Xie, X. Zhao, C. F. Guo, Graded intrafillable architecture-based iontronic pressure sensor with ultra-broad-range high sensitivity. *Nat. Commun.* **11**, 209 (2020).

14. C. M. Boutry, L. Beker, Y. Kaizawa, C. Vassos, H. Tran, A. C. Hinckley, R. Pfattner, S. Niu, J. Li, J. Claverie, Z. Wang, J. Chang, P. M. Fox, Z. Bao, Biodegradable and flexible arterial-pulse sensor for the wireless monitoring of blood flow. *Nat. Biomed. Eng.* **3**, 47–57 (2019).

15. S. C. B. Mannsfeld, B. C.-K. Tee, R. M. Stoltenberg, C. V. H.-H Chen, S. Barman, B. V. O. Muir, A. N. Sokolov, C. Reese, Z. Bao, Highly sensitive flexible pressure sensors with microstructured rubber dielectric layers. *Nat. Mater.* **9**, 859–864 (2010).

16. C. Majidi, Soft sensors that can feel it all. *Sci. Robot.* **5**, eabf0894 (2020).

17. D. Kwon, T. I. Lee, J. Shim, S. Ryu, M. S. Kim, S. Kim, T.S. Kim, I. Park, Highly sensitive, flexible, and wearable pressure sensor based on a giant piezocapacitive effect of three-dimensional microporous elastomeric dielectric layer. *ACS Appl. Mater. Interfaces* **8**, 16922–16931 (2016).

18. C. Keplinger, J. Y. Sun, C. C. Foo, P. Rothemund, G. M. Whitesides, Z. Suo, Stretchable, transparent, ionic conductors. *Science* **341**, 984–987 (2013).

19. J. Y. Sun, C. Keplinger, G. M. Whitesides, Z. Suo, Ionic skin. *Adv. Mater.* **26**, 7608–7614 (2014).

20. Q. Su, X. Huang, K. Lan, T. Xue, W. Gao, Q. Zou, Highly sensitive ionic pressure sensor based on concave meniscus for electronic skin. *J. Micromech. Microeng.* **30**, 015009 (2020).

21. P. Lu, L. Wang, P. Zhu, J. Huang, Y. Wang, N. Bai, Y. Wang, G. Li, J. Yang, K. Xie, J. Zhang, B. Yu, Y. Dai, C. F. Guo, Iontronic pressure sensor with high sensitivity and linear response over a wide pressure range based on soft micropillared electrodes. *Sci. Bull.* **66**, 1091–1100 (2021).

22. Z. Song, T. Ma, R. Tang, Q. Cheng, X. Wang, D. Krishnaraju, R. Panat, C. K. Chan, H. Yu, H. Jiang, Origami lithium-ion batteries. *Nat. Commun.* **5**, 3140 (2014).

23. K. I. Jang, H. U. Chung, S. Xu, C. H. Lee, H. Luan, J. Jeong, H. Cheng, G.T. Kim, S. Y. Han, J. W. Lee, J. Kim, M. Cho, F. Miao, Y. Yang, H. N. Jung, M. Flavin, H. Liu, G. W. Kong, K. J. Yu, S. I. Rhee, J. Chung, B. Kim, J. W. Kwak, M. H. Yun, J. Y. Kim, Y. M. Song, U. Paik, Y. Zhang, Y. Huang, J. A. Rogers, Soft network composite materials with deterministic and bio-inspired designs. *Nat. Commun.* **6**, 6566 (2015).

24. Y. Zhang, J. Yang, X. Hou, G. Li, L. Wang, N. Bai, M. Cai, L. Zhao, Y. Wang, J. Zhang, K. Chen, X. Wu, C. Yang, Y. Dai, Z. Zhang, C. F. Guo, Highly stable flexible pressure sensors with a quasi-homogeneous composition and interlinked interfaces. *Nat. Commun.* **13**, 1317 (2022).

25. Z. Shen, X. Zhu, C. Majidi, G. Gu, Cutaneous Ionogel mechanoreceptors for soft machines, physiological sensing, and amputee prostheses. *Adv. Mater.* **33**, e2102069 (2021).

26. H. Hu, D. Wang, H. Tian, Q. Huang, C. Wang, X. Chen, Y. Gao, X. Li, X. Chen, Z. Zheng, J. Shao, Bioinspired hierarchical structures for contact□sensible adhesives. *Adv. Funct. Mater.* **32**, e2109076 (2021).

27. Y. Zhu, L. Lin, Y. Chen, Y. Song, W. Lu, Y. Guo, A self-healing, robust adhesion, multiple stimuli-response hydrogel for flexible sensors. *Soft Matter* **16**, 2238–2248 (2020).

28. Q. Wang, H. Ding, X. Hu, X. Liang, M. Wang, Q. Liu, Z. Li, G. Sun, A dual-trigger-mode ionic hydrogel sensor for contact or contactless motion recognition. *Mater. Horiz.* **7**, 2673–2682 (2020).

29. Z. Li, S. Zhang, Y. Chen, H. Ling, L. Zhao, G. Luo, X. Wang, M. C. Hartel, H. Liu, Y. Xue, R. Haghniaz, K. J. Lee, W. Sun, H. J. Kim, J. Lee, Y. Zhao, Y. Zhao, S. Emaminejad, S. Ahadian, N. Ashammakhi, M. R. Dokmeci, Z. Jiang, A. Khademhosseini, Gelatin methacryloyl-based tactile sensors for medical wearables. *Adv. Funct. Mater.* **30**, 2003601 (2020).

30. A. Chortos, Z. Bao, Skin-inspired electronic devices. *Mater. Today* **17**, 321–331 (2014).

31. B. Nie, R. Li, J. D. Brandt, T. Pan, Iontronic microdroplet array for flexible ultrasensitive tactile sensing. *Lab Chip* **14**, 1107–1116 (2014).

32. W. Xiong, D. Guo, Z. Yang, C. Zhu, Y. Huang, Conformable, programmable and step-linear sensor array for large-range wind pressure measurement on curved surface. *Sci. China Technol. Sci.* **63**, 2073–2081 (2020).

33. Z. Yan, L. Wang, Y. Xia, R. Qiu, W. Liu, M. Wu, Y. Zhu, S. Zhu, C. Jia, M. Zhu, R. Cao, Z. Li, X. Wang, Flexible high□resolution triboelectric sensor array based on patterned laser□induced graphene for self□powered real□time tactile sensing. *Adv. Funct. Mater.* **31**, e2100709 (2021).

34. S. Sharma, A. Chhetry, S. Zhang, H. Yoon, C. Park, H. Kim, M. Sharifuzzaman, X. Hui, J. Y. Park, Hydrogen-bond-triggered hybrid nanofibrous membrane-based wearable pressure sensor with ultrahigh sensitivity over a broad pressure range. *ACS Nano* **15**, 4380–4393 (2021).

35. T. Jin, Z. Sun, L. Li, Q. Zhang, M. Zhu, Z. Zhang, G. Yuan, T. Chen, Y. Tian, X. Hou, C. Lee, Triboelectric nanogenerator sensors for soft robotics aiming at digital twin applications. *Nat. Commun.* **11**, 5381 (2020).

36. Z. Sun, M. Zhu, X. Shan, C. Lee, Augmented tactile-perception and haptic-feedback rings as human-machine interfaces aiming for immersive interactions. *Nat. Commun.* **13**, 5224 (2022).

37. M. Zhu, Z. Sun, Z. Zhang, Q. Shi, T. He, H. Liu, T. Chen, C. Lee, Haptic-feedback smart glove as a creative human-machine interface (HMI) for virtual/augmented reality applications. *Sci. Adv*. **6**, eaaz8693 (2020).

38. P. Che, X. Han, P. Guo, X. Wang, S. Cheng, K. Han, L. Jiang, L. Heng, Robust yet flexible slippery layered composite surfaces with a programmable pressure-resistance response under extreme environmental conditions. *J. Mater. Chem. A* **10**, 14933–14942 (2022).

39. W. Cheng, J. Wang, Z. Ma, K. Yan, Y. Wang, H. Wang, S. Li, Y. Li, L. Pan, Y. Shi, Flexible pressure sensor with high sensitivity and low hysteresis based on a hierarchically microstructured electrode. *IEEE Electron. Device Lett.* **39**, 288–291 (2018).

40. Y. Yang, M. Zhou, J. Peng, X. Wang, Y. Liu, W. Wang, D. Wu, Robust, anti-freezing and conductive bonding of chitosan-based double-network hydrogels for stable-performance flexible electronic. *Carbohydr. Polym.* **276**, 118753 (2022).

41. F. Mo, Y. Huang, Q. Li, Z. Wang, R. Jiang, W. Gai, C. Zhi, A highly stable and durable capacitive strain sensor based on dynamically super□tough hydro/organo□gels. *Adv. Funct. Mater.* **31**, 2010830 (2021).

42. T. P. Nguyen, B. M. Tran, N. Y. Lee, Thermally robust and biomolecule-friendly room-temperature bonding for the fabrication of elastomer-plastic hybrid microdevices. *Lab Chip* **16**, 3251–3259 (2016).

43. S. Kuddannaya, Y. J. Chuah, M. H. A. Lee, N. V. Menon, Y. Kang, Y. Zhang, Surface chemical modification of poly(dimethylsiloxane) for the enhanced adhesion and proliferation of mesenchymal stem cells. *ACS Appl. Mater. Interfaces* **5**, 9777–9784 (2013).

44. N. N. R. Ahmad, H. Mukhtar, D. F. Mohshim, R. Nasir, Z. Man, Surface modification in inorganic filler of mixed matrix membrane for enhancing the gas separation performance. *Rev. Chem. Eng.* **32**, 181–200 (2016).

45. J. J. Senkevich, C. J. Mitchell, G.-R. Yang, T.-M. Lu, Surface chemistry of mercaptan and growth of pyridine short-chain alkoxy silane molecular layers. *Langmuir* **18**, 1587–1594 (2002).

46. Y. Zhang, C. J. Sheehan, J. Zhai, G. Zou, H. Luo, J. Xiong, Y. T. Zhu, Q. X. Jia, Polymer-embedded carbon nanotube ribbons for stretchable conductors. *Adv. Mater.* **22**, 3027–3031 (2010).

47. C. Creton, M. Ciccotti, Fracture and adhesion of soft materials: A review. *Rep. Prog. Phys.* **79**, 046601 (2016).

48. H. Yuk, T. Zhang, S. Lin, G. A. Parada, X. Zhao, Tough bonding of hydrogels to diverse non-porous surfaces. *Nat. Mater.* **15**, 190–196 (2016).

49. Z. Wang, C. Xiang, X. Yao, P. le Floch, J. Mendez, Z. Suo, Stretchable materials of high toughness and low hysteresis. *Proc. Natl. Acad. Sci. U.S.A.* **116**, 5967–5972 (2019).

50. H. Yao, W. Yang, W. Cheng, Y. J. Tan, H. H. See, S. Li, H. P. A. Ali, B. Z. H. Lim, Z. Liu, B. C. K. Tee, Near-hysteresis-free soft tactile electronic skins for wearables and reliable machine learning. *Proc. Natl. Acad. Sci. U.S.A.* **117**, 25352–25359 (2020).
